# More than 75 percent decline over 27 years in total flying insect biomass in protected areas

**DOI:** 10.1371/journal.pone.0185809

**Published:** 2017-10-18

**Authors:** Caspar A. Hallmann, Martin Sorg, Eelke Jongejans, Henk Siepel, Nick Hofland, Heinz Schwan, Werner Stenmans, Andreas Müller, Hubert Sumser, Thomas Hörren, Dave Goulson, Hans de Kroon

**Affiliations:** 1 Radboud University, Institute for Water and Wetland Research, Animal Ecology and Physiology & Experimental Plant Ecology, PO Box 9100, 6500 GL Nijmegen, The Netherlands; 2 Entomological Society Krefeld e.V., Entomological Collections Krefeld, Marktstrasse 159, 47798 Krefeld, Germany; 3 University of Sussex, School of Life Sciences, Falmer, Brighton BN1 9QG, United Kingdom; University of Saskatchewan, CANADA

## Abstract

Global declines in insects have sparked wide interest among scientists, politicians, and the general public. Loss of insect diversity and abundance is expected to provoke cascading effects on food webs and to jeopardize ecosystem services. Our understanding of the extent and underlying causes of this decline is based on the abundance of single species or taxonomic groups only, rather than changes in insect biomass which is more relevant for ecological functioning. Here, we used a standardized protocol to measure total insect biomass using Malaise traps, deployed over 27 years in 63 nature protection areas in Germany (96 unique location-year combinations) to infer on the status and trend of local entomofauna. Our analysis estimates a seasonal decline of 76%, and mid-summer decline of 82% in flying insect biomass over the 27 years of study. We show that this decline is apparent regardless of habitat type, while changes in weather, land use, and habitat characteristics cannot explain this overall decline. This yet unrecognized loss of insect biomass must be taken into account in evaluating declines in abundance of species depending on insects as a food source, and ecosystem functioning in the European landscape.

## Introduction

Loss of insects is certain to have adverse effects on ecosystem functioning, as insects play a central role in a variety of processes, including pollination [1, 2], herbivory and detrivory [3, 4], nutrient cycling [4] and providing a food source for higher trophic levels such as birds, mammals and amphibians. For example, 80% of wild plants are estimated to depend on insects for pollination [2], while 60% of birds rely on insects as a food source [5]. The ecosystem services provided by wild insects have been estimated at $57 billion annually in the USA [6]. Clearly, preserving insect abundance and diversity should constitute a prime conservation priority.

Current data suggest an overall pattern of decline in insect diversity and abundance. For example, populations of European grassland butterflies are estimated to have declined by 50% in abundance between 1990 and 2011 [7]. Data for other well-studied taxa such as bees [8–14] and moths [15–18] suggest the same trend. Climate change, habitat loss and fragmentation, and deterioration of habitat quality have been proposed as some of the prime suspects responsible for the decline [9–11, 13, 18–22]. However, the number of studies on insect trends with sufficient replication and spatial coverage are limited [10, 23–25] and restricted to certain well-studied taxa. Declines of individual species or taxa (e.g. [7, 26]) may not reflect the general state of local entomofauna [27]. The total insect biomass would then be a better metric for the status of insects as a group and its contribution to ecosystem functioning, but very few studies have monitored insect biomass over an extensive period of time [28]. Hence, to what extent total insect biomass has declined, and the relative contribution of each proposed factor to the decline, remain unresolved yet highly relevant questions for ecosystem ecology and conservation.

Here, we investigate total aerial insect biomass between 1989 and 2016 across 96 unique location-year combinations in Germany, representative of Western European low-altitude nature protection areas embedded in a human-dominated landscape (S1 Fig). In all years we sampled insects throughout the season (March through October), based on a standardized sampling scheme using Malaise traps. We investigated rate of decline in insect biomass, and examined how factors such as weather, habitat and land use variables influenced the declines. Knowledge on the state of insect biomass, and it’s direction over time, are of broad importance to ecology and conservation, but historical data on insect biomass have been lacking. Our study makes a first step into filling this gap, and provides information that is vital for the assessment of biodiversity conservation and ecosystem health in agricultural landscapes.

## Materials and methods

### Data

#### Biomass data

Biomass data were collected and archived using a standardized protocol across 63 unique locations between 1989 and 2016 (resulting in 96 unique location-year combinations) by the Entomological Society Krefeld. The standardized protocol of collection has been originally designed with the idea of integrating quantitative aspects of insects in the status assessment of the protected areas, and to construct a long-term archive in order to preserve (identified and not-identified) specimens of local diversity for future studies. In the present study, we consider the total biomass of flying insects to assess the state of local entomofauna as a group.

**Fig 1:**
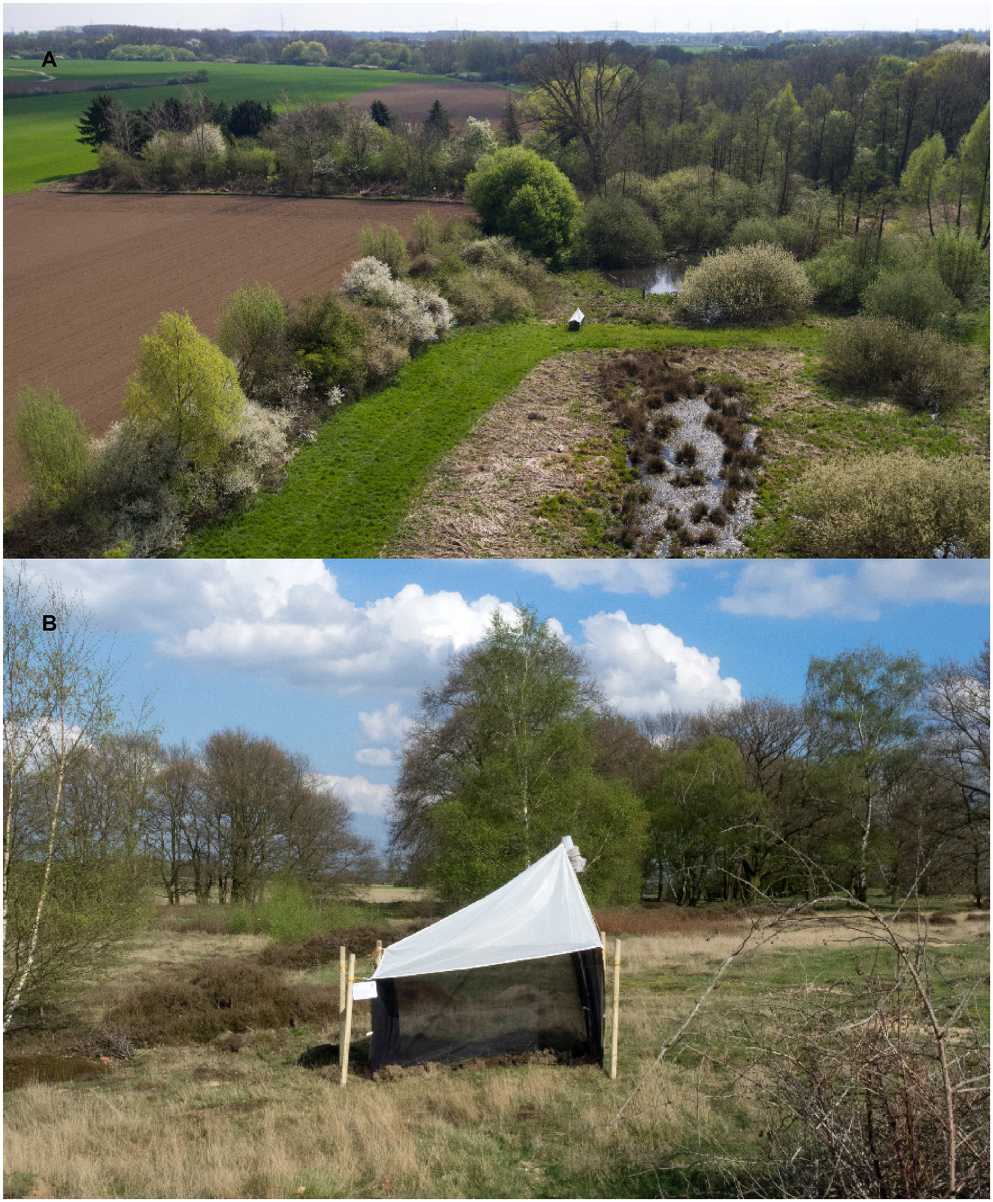
Examples of operating malaise traps in protected areas in western Germany, in habitat cluster 1 (A) and cluster 2 (B) (see Materials and methods).

All trap locations were situated in protected areas, but with varying protection status: 37 locations are within Natura2000 sites, seven locations within designated Nature reserves, nine locations within Protected Landscape Areas (with funded conservation measures), six locations within Water Protection Zones, and four locations of protected habitat managed by Regional Associations. For all location permits have been obtained by the relevant authorities, as listed in the S1 Appendix. In our data, traps located in nutrient-poor heathlands, sandy grasslands, and dune habitats provide lower quantities of biomass as compared to nutrient nutrient-rich grasslands, margins and wastelands. As we were interested in whether the declines interact with local productivity, traps locations were pooled into 3 distinct habitat clusters, namely: nutrient-poor heathlands, sandy grassland, and dunes (habitat cluster 1, n = 19 locations, Fig 1A), nutrient-rich grasslands, margins and wasteland (habitat cluster 2, n = 41 locations, Fig 1B) and a third habitat cluster that included pioneer and shrub communities (n = 3 locations).

Most locations (59%, n = 37) were sampled in only one year, 20 locations in two years, five locations in three years, and one in four years, yielding in total 96 unique location-year combinations of measurements of seasonal total flying insect biomass. Our data do not represent longitudinal records at single sites, suitable to derive location specific trends (e.g. [28]). Prolonged trapping across years is in the present context (protected areas) deemed undesirable, as the sampling process itself can negatively impact local insect stocks. However, the data do permit an analysis at a higher spatial level, i.e. by treating seasonal insect biomass profiles as random samples of the state of entomofauna in protected areas in western Germany.

Malaise traps were deployed through the spring, summer and early autumn. They operated continuously (day and night), and catches were emptied at regular intervals, on average every 11.2 days (sd = 6.3). We collected in total 1503 trap samples, with an average of 16 (4–35) successive catches per location-year combination (Table 1). Between 1989 and 2016, a total of 53.54kg of invertebrates have been collected and stored, over a total trap exposure period of 16908 days, within an average of 176 exposure days per location-year combination. Malaise traps are known to collect a much wider diversity of insect species (e.g. [29–31]) as compared to suction traps (e.g. [28]) and are therefore considered superior as a method of collecting flying insects. On the basis of partial assessments, we can assume that the total number of insects included in 53.54 kg biomass represents millions of individuals.

**Table 1:** Overview of malaise-trap samples sizes. For each year, the number of locations sampled, the number of location re-sampled, total number of samples, as well as mean and standard deviation of exposure time at the trap locations (in days) are presented.

Year	Number of locations	Number of locations sampled previously	Number of Samples	Mean exposure time	St. Dev exposure time
1989	8	0	162	146.62	12.81
1990	2	0	62	228.50	34.65
1991	1	0	10	146.00	
1992	4	0	54	118.75	15.50
1993	4	0	39	109.50	59.74
1994	4	0	60	170.75	72.83
1995	2	0	41	144.00	93.34
1997	1	0	20	162.00	
1999	2	0	56	196.00	0.00
2000	2	1	47	174.00	11.31
2001	3	2	81	190.00	0.00
2003	3	1	80	201.00	7.81
2004	2	0	48	200.00	5.66
2005	4	0	70	198.75	30.53
2006	2	0	26	188.00	0.00
2007	2	0	15	192.00	0.00
2008	2	0	24	162.00	0.00
2009	4	0	23	120.50	2.89
2010	2	0	12	85.00	0.00
2011	1	0	4	68.00	
2012	2	0	23	158.50	4.95
2013	8	2	126	175.50	21.71
2014	23	19	348	212.74	11.21
2015	1	1	10	224.00	
2016	7	7	62	190.86	12.56

The sampling was standardized in terms of trap construction, size and design (identical parts), colors, type of netting and ground sealing, trap orientation in the field as well as slope at the trap location. Hence none of the traps differed in any of these field aspects. Our trap model was similar to the bi-colored malaise trap model by Henry Townes [32, 33]. The traps, collecting design, and accompanying methods of biomass measurement as designed and applied by the Entomological Society Krefeld are described elsewhere [34–36] and in S2 Appendix.

Trap catches were stored in 80% ethanol solution, prior to weighing, and total insect biomass of each catch (bottle) was obtained based on a standardized measurement protocol by first subtracting fluid content. In order to optimally preserve samples for future species determination, the insects were weighed in an alcohol-wet state. First, the alcohol concentration in the vessels was stabilized to 80%, while this concentration was controlled with an areometer over a period of at least two days. In order to obtain biomass per sample with sufficient accuracy and comparability, the measuring process was fixed using a standardized protocol [34]. For this purpose the insects of a sample were poured onto a stainless steel sieve (10cm diameter) of 0.8 mm mesh width. This sieve is placed slightly obliquely (30 degrees) over a glass vessel. The skew position accelerates the first runoff of alcohol and thus the whole measuring procedure. The drop sequence is observed with a stopwatch. When the time between two drops has reached 10 seconds for the first time, the weighing process is performed with a laboratory scale. For the determination of the biomass, precision scales and analytical scales from Mettler company were used with an accuracy of at least 0.1g and controlled with calibrated test weights at the beginning of a new weighing series. In a series of 84 weightings of four different samples repeating this measurement procedure, an average deviation from the mean value of the measurement results of 0.4 percent was observed (unpublished results).

#### Weather data

Climate change is a well-known factor responsible for insect declines [15, 18, 21, 37]. To test if weather variation could explain the observed decline, we included mean daily temperature, precipitation and wind speed in our analysis, integrating data from 169 weather stations [38] located within 100km to the trap locations. We examined temporal trends in each weather variable over the course of the study period to assess changes in climatic conditions, as a plausible explanation for insect decline. Estimates of each weather variable at the trap locations were obtained by interpolation of each variable from the 169 climate stations.

We initially considered mean daily air temperature, precipitation, cloud cover, relative air moisture content, wind speed, and sunshine duration. However, only temperature, precipitation and wind speed were retained for analysis, as the other variables were significantly correlated with the selected variables [R(temperature, cover) = −43.2%, R(temperature, sunshine) = 53.4%, R(precipitation, moisture) = −47.3%] and because we wanted to keep the number of covariates as low as possible. Additionally, we calculated the number of frost days and the sum of precipitation in the months November- February preceding a sampling season. We used spatio-temporal geostatistical models [39, 40] to predict daily values for each weather variable to each trap location. Amongst other methods, the geostatistical approach is considered a superior interpolation method in order to derive weather variables to trap locations [41]. Uncertainty in interpolated variables such as wind speed is usually associated with altitude differences. However, as our trap locations are all situated in lowland areas with little altitude variation, we do not expect a large error in our interpolations at trap locations.

We decomposed the daily values of each weather variable into a long-term average trend (between years), a mean seasonal trend, and a yearly seasonal anomaly component (S2 Fig), modeled using regression splines [42] while controlling for altitude of weather stations. The remaining residual daily values of each station were further modeled using a spatio-temporal covariance structure. For example, temperature *T*, on given day *t*, of a given year *k* at a given trap location *s* is modeled as:

$$\begin{array}{c} T\left(t,s,k\right)=f_{k}\left(k\right)+f_{t}\left(t\right)+r\left(k,t\right)+a\times h+C_{s,t} \end{array}$$ (1)

where *f*_*k*_(*k*) is the long-term trend over the years (a thin plate regression spline), *f*_*t*_(*t*) the mean seasonal trend within years (a penalized cyclic cubic regression spline), *r*(*k*, *t*) the mean residual seasonal component, which measures annual anomaly in mean daily values across selected stations, and *a* is the linear coefficient for the altitude *h* effect. The spatio-temporal covariance structure *C*_*s*, *t*_, fitted independently to the residuals of each weather variable model, allowed us to deal with lack of independence between daily weather data within and between stations, as well as to interpolate to trap locations using kriging. Altitude of trap locations was extracted from a digital elevation models at 90m resolution [43].

#### Land use data

Land use variables (and changes therein) were derived from aerial photographs [44] taken within two distinct time periods (between 1989–1994, and between 2012–2015), and allowed us to characterize land use composition at surroundings of the traps, as well as changes over time. We distinguished cover of forests, agricultural areas, natural grassland, and surface water. For each trap location, aerial photographs were manually processed, polygons extracted and categorized, and their surface area calculated with a radius of 200 meter. Preliminary analysis of the relationship between log biomass and landuse variables, on a subset of the trap locations, indicated that land use elements at 200m radius better predicted insect biomass than elements at 500 and 1000m radius, similar to findings elsewhere for wild bees [45]. Land use variables were measured at a coarse temporal resolution, but fortunately cover the temporal span of insect sampling. To link the cover of a given land use variable to the insect biomass samples in a particular year, we interpolated coverage between the two time points to the year of insect sampling using generalized linear models with a binomial error distribution, a logit link, and an estimated dispersion parameter. Mean distributions of land use at each of the two time points are depicted in S3A & S3B Fig.

#### Habitat data

Plant inventories were conducted in the immediate surroundings (within 50m) of the trap, in the same season of insect sampling. These data permitted the assessment of plant species richness (numbers of herbs, shrubs and trees) and environmental conditions based on average Ellenberg values [46–48], as well as changes therein over time. Each Ellenberg indicator (we considered nitrogen, pH, light, temperature and moisture) was averaged over all species for each location-year combination. We examined annual trends in each of the above-mentioned variables in order to uncover potential structural changes in habitat characteristics over time. Species richness was analyzed using mixed-effects generalized linear models [49] with a random intercept for trap location and assuming a Poisson distribution for species richness, and a normal distribution for mean Ellenberg indicator values. Although a Poisson distribution fitted tree and shrub species adequately, (residual deviance/ degree of freedom = 0.94 and 1.04 respectively), severe overdispersion was found for herb species richness (residual deviance/ degree of freedom = 2.16). Trend coefficients of richness over time between a Poisson mixed effects model and a negative binomial model were comparable but differed in magnitude (Poisson GLMM: −0.034 (se = 0.003), vs NB GLMM −0.027 (se = 0.006)). Although the fit is not perfect in the case of herb richness, we believe our trend adequately describes direction of change over time. Mean changes in plant species richness are depicted in S3C Fig.

### Insect biomass model

The temporal resolution of the trap samples (accumulated over several days) is not directly compatible with the temporal distribution of the weather data (daily values). Additionally, variable exposure intervals between trap samples is expected to induce variation in trapped biomass between samples, and hence induce heteroscedasticity. Furthermore, biomass data can numerically only be positive on the real line, and we require a model to reflect this property of the data. Because of the unequal exposure intervals however, log-transforming the response would be inappropriate, because we require the sum of daily values after exponentiation, rather that the exponent of the sum of log-daily biomass values. In order to indirectly relate biomass to daily weather variables, to account for the variation in time exposure intervals over which biomass was accumulated in the samples, and to respect the non-negative nature of our data, we modeled the biomass of each catch as the sum of the expected (but unobserved) latent daily biomass. The mass *m* of each sample *j*, at site *s* in year *k*, is assumed to be distributed normally about the sum of the latent expected daily mass (*z*_*t*, *s*, *k*_), with variance $\sigma_{j}^{2}$:

$$\begin{array}{c} m_{j,s,k}∼N\left(\mu_{j,s,k},\sigma_{j}^{2}\right) \end{array}$$ (2)

subject to $\mu_{j,s,k}=\sum_{t=\tau_{1}\left(j\right)}^{\tau_{2}\left(j\right)}z_{t,s,k}$ where *τ*_1_ and *τ*_2_ mark the exposure interval (in days) of biomass collection of each sample *j*. The latent daily biomass itself is represented by a log normal distribution, in which coefficients for covariates, random effects and residual variance are all represented on the log scale. In turn, daily biomass is modeled as

$$\begin{array}{c} z_{t,s,k}=e^{y_{t,s,k}} \end{array}$$ (3)

$$\begin{array}{c} y_{t,s,k}=c+log\left(\lambda \right)k+X\beta_{x}+u_{s} \end{array}$$ (4)

where *c* is a global intercept, **X** a design matrix of dimensions n×p (number of samples × number of covariates; see Model analysis below), *β*_*x*_ the corresponding vector of coefficients that measure the weather, habitat and land use effects, and *log*(*λ*) a mean annual population growth rate parameter. The random term (*u*_*s*_) denotes the location-specific random effect assumed to be distributed normally about zero $u_{s}∼N\left(0,\sigma_{site}^{2}\right)$. The exponentiation of the right hand side of Eq (3) ensures expected values to be positive.

The expected residual variance of each sample $\sigma_{j}^{2}$, is expressed as the sum of variances of daily biomass values ($\sigma_{t,s,k}^{2}$).

$$\begin{array}{c} \sigma_{j}^{2}=\sum_{t=\tau_{1}\left(j\right)}^{\tau_{2}\left(j\right)}\sigma_{t,s,k}^{2} \end{array}$$ (5)

The variances of daily biomass should respect the non-negative nature of the data as well. Additionally, we are interested in being able to compare the residual variance with the random effects variance, and this requires them to be on the same scale. Therefore, we expressed the variance of the daily biomass as a function of the variance of the logarithm of the daily biomass. Using the method of moments:

$$\begin{array}{c} \sigma_{t,s,k}^{2}=e^{2y_{t,s,k}+v}\left(e^{v}-1\right) \end{array}$$ (6)

where *v* represents the residual variance of daily log-biomass.

### Analysis

We developed a series of models each consisting of a set of explanatory variables that measure aspects of climate, land use and local habitat characteristics. Significant explanatory variables in these models were combined into a final model, which was then reduced to exclude insignificant effects. An overview of which covariates were included in each model is given in Table 2.

**Table 2:** Overview of covariates included in each of the seven models. The year covariate yields the annual trend coefficient.

Covariate class	Covariate name	Null model	Basic	Weather	Habitat	Land use Interactions	Land use+	Final model
Temporal	Intercept	✔	✔	✔	✔	✔	✔	✔
	Day number	✔	✔	✔	✔	✔	✔	✔
	Day number^2^	✔	✔	✔	✔	✔	✔	✔
	Year		✔	✔	✔	✔	✔	✔
Climate	Temperature			✔				✔
	Precipitation			✔				✔
	Wind Speed			✔				
	Frost days			✔				✔
	Winter Precipitation			✔				
Habitat	Herb Species				✔			✔
	Tree Species				✔			✔
	Nitrogen				✔			
	pH				✔			
	Moisture				✔			
	Light				✔			✔
	Ellen. Temperature				✔			✔
	Habitat cluster 2	✔	✔	✔	✔	✔	✔	
	Habitat cluster 3	✔	✔	✔	✔	✔	✔	
Landscape	Arable land					✔	✔	✔
	Grassland					✔	✔	✔
	Forest					✔	✔	✔
	Water					✔	✔	✔
Interactions	Year × Day number		✔	✔	✔	✔	✔	✔
	Year × Day number^2^		✔	✔	✔	✔	✔	✔
	Year × Agriculture						✔	✔
	Year × Forest						✔	✔
	Year × Water						✔	
	Year × Grassland						✔	✔
Variance	*σ*_*site*_	✔	✔	✔	✔	✔	✔	✔
	*v*	✔	✔	✔	✔	✔	✔	✔

Weather effects explored were daily temperature, precipitation and wind speed, as well as the number of frost days and sum of precipitation in the preceding winter. Habitat effects explored tree and herb species richness, as well as average Ellenberg values for nitrogen, pH, light, temperature and moisture, per location-year combination. Land use effects explored the fractions of agricultural area, forest, grass, and surface water in a radius of 200m around the plot location.

Parameter values are obtained by the use of Markov chain Monte Carlo (MCMC) methods by the aid of JAGS (Just Another Gibbs Sampler [50]) invoked through R [51] and the R2Jags package [52]. JAGS model scripts are given in S1 Code, while data are given in S1 and S2 Dataset. For each model, we ran 3 parallel chains each consisting of 24000 iterations (first 4000 discarded), and kept every 10^*th*^ value as a way to reduce within chain autocorrelation. We used vague priors for all parameters, with uniform distributions for the residual and random effect variance components, and flat normal distributions (with very high variance) for all other parameters. Covariates in **X** were standardized prior to model fitting, with the exception of year (values 1–26), and land use variables (proportions within 0–1 range).

For all models, we computed the Deviance Information Criterion [53] (DIC) as well as the squared correlation coefficient (R^2^) between observed and mean posterior estimates of biomass on the log scale. Results are given in Table 3. Parameter convergence was assessed by the potential scale reduction factor [54] (commonly $\hat{R}$), that measures the ratio of posterior distributions between independent MCM chains (in all models, all parameters attained values below 1.02). For all models, we confirmed that the posterior distribution of the trend coefficient did not confound any other variable by plotting the relevant posterior samples and computing pairwise correlation coefficients.

**Table 3:** Results for 7 models ranked by Deviance Information Criterion (DIC). For each model, the number of parameters, the Deviance Information Criterion, the effective number of parameters (pD), calculated *R*^2^ and difference in DIC units between each model and the model with lowest ΔDIC. See Table 2 for covariates included in each model.

model	npar	Deviance	DIC	pD	R^2^	ΔDIC
Final	23	12082.48	12177.07	94.59	0.67	0.00
Weather	13	12178.84	12261.52	82.68	0.65	84.45
Land use+ Interactions	16	12336.22	12427.16	90.95	0.62	250.09
Habitat	15	12354.95	12445.93	90.98	0.62	268.86
Land use	12	12377.27	12453.23	75.97	0.61	276.16
Basic	8	12390.26	12465.08	74.82	0.61	288.00
Null	5	13230.65	13307.59	76.94	0.39	1130.52

Our basic model included habitat cluster (3 levels), a quadratic effect for day number, an annual trend coefficient measuring the rate of biomass change, and the interactions between the annual trend coefficient and the day number variables. Next we developed 3 models each consisting of either weather variables (S1 Table), land use variables (S2 Table), or habitat variables. Because interactions between the annual rate of change and land use variables seemed plausible, a fourth model was developed to include these interactions (S3 Table). Finally, all significant variables were combined into our final model (Table 4), which included effects of an annual trend coefficient, season (linear and quadratic effect of day number), weather (temperature, precipitation, number of frost days), land use (cover of grassland and water, as well as interaction between grassland cover and trend), and habitat (number of herb and tree species as well as Ellenberg temperature).

**Table 4:** Posterior parameter estimates of the final mixed effects model of daily insect biomass. For each included variable, the corresponding coefficient mean, standard deviation and 95% credible intervals are given. P-values were calculated empirically based on posterior distributions of coefficients.

Class	Variable	mean	sd	2.50%	97.50%	P	
Temporal	Intercept	2.450	0.233	1.983	2.891	0.000	***
	log(*λ*)	-0.080	0.007	-0.094	-0.067	0.000	***
	Day number	-0.100	0.028	-0.155	-0.045	0.001	***
	Day number^2^	-0.447	0.029	-0.504	-0.392	0.000	***
Weather	Temperature	0.304	0.022	0.263	0.347	0.000	***
	Precipitation	-0.071	0.034	-0.143	-0.009	0.014	*
	Frost days	-0.021	0.024	-0.067	0.025	0.194	
Land use	Habitat Cluster 2	0.420	0.162	0.080	0.729	0.007	**
	Habitat Cluster 3	0.332	0.237	-0.133	0.806	0.078	.
	Arable land	-1.063	0.184	-1.420	-0.709	0.000	***
	Forest	-0.522	0.216	-0.947	-0.121	0.007	**
	Grassland	0.819	0.233	0.367	1.265	0.000	***
	Water	-0.327	0.170	-0.659	0.005	0.027	*
Habitat	Herb species	-0.054	0.045	-0.137	0.037	0.119	
	Tree Species	0.104	0.032	0.041	0.167	0.000	***
	Ell. Nitrogen	0.181	0.065	0.051	0.311	0.003	**
	Ell. Light	0.162	0.039	0.088	0.236	0.000	***
	Ell. Temperature	-0.071	0.031	-0.131	-0.011	0.010	**
Intercations	Year × Day number	-0.003	0.001	-0.006	-0.000	0.017	*
	Year × Day number^2^	0.010	0.001	0.007	0.013	0.000	***
	Year × Arable land	0.047	0.008	0.031	0.064	0.000	***
	Year × Forest	0.035	0.010	0.016	0.055	0.000	***
	Year × Grassland	-0.059	0.014	-0.086	-0.033	0.000	***
Random effects	*σ*_*site*_	0.334	0.037	0.270	0.412		
Residual variation	*v*	0.870	0.009	0.852	0.889		

Our estimate of decline is based on our basic model, from which we can derive seasonal estimates of daily biomass for any given year. The basic model includes only a temporal (annual and seasonal effects, as well as interactions) and a basic habitat cluster distinction (additive effects only) as well as a random trap location effect. We here report the annual trend coefficient, as well as a weighted estimate of decline that accounts for the within season differences in biomass decline. The weighted insect biomass decline was estimated by projecting the seasonal biomass (1-April to 30-October) for years 1989 and 2016 using coefficients our basic model, and then dividing the summed (over the season) biomass of 2016 by the summed biomass over 1989.

Using our final model, we assessed the relative contribution (i.e. net effect) of the explanatory variables to the observed decline, both combined and independently. To this aim we projected the seasonal daily biomass for the years 1989 and 2016 twice: first we kept covariates at their mean values during the early stages of the study period, and second we allowed covariate values to change according to the observed mean changes (see S2 and S3 Figs). Difference in the total biomass decline between these two projections are interpreted as the relative contribution of the explanatory variables to the decline. The marginal (i.e. independent) effects of each covariate were calculated by projecting biomass increase/decline as result of the observed temporal developments in each variable separately, and expressing it as percentual change.

Our data provide repetitions across years for only a subset of locations (n = 26 out of 63). As such, spatial variation in insect biomass may confound the estimated trend. To verify that this is not the case, we fitted our basic model (but excluding the day number and year interaction to avoid overparameterization) to the subset of our data that includes only locations that were sampled in more than one year. Seasonal profiles of daily biomass values are depicted in S4 Fig. Finally, we reran our basic model for the two (of the three) habitat clusters (for which sufficient data existed; see Biomass Data) separately in order to compare the rate of decline between them (S5 Fig).

## Results

**Fig 2:**
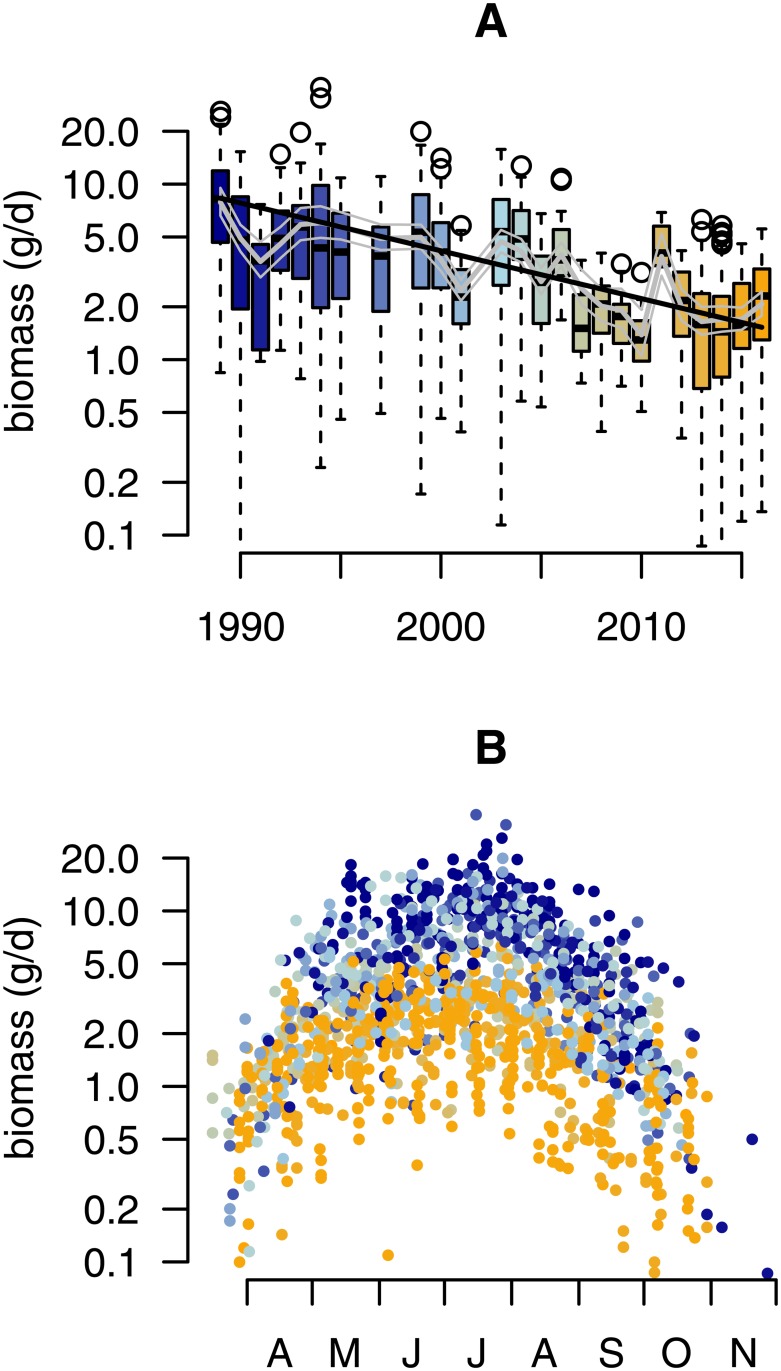
Temporal distribution of insect biomass. (A) Boxplots depict the distribution of insect biomass (gram per day) pooled over all traps and catches in each year (n = 1503). Based on our final model, the grey line depicts the fitted mean (+95% posterior credible intervals) taking into account weather, landscape and habitat effects. The black line depicts the mean estimated trend as estimated with our basic model. (B) Seasonal distribution of insect biomass showing that highest insect biomass catches in mid summer show most severe declines. Color gradient in both panels range from 1989 (blue) to 2016 (orange).

**Fig 3:**
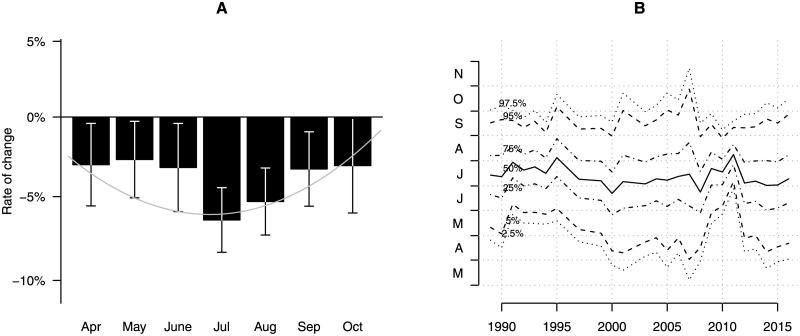
Seasonal decline and phenology. (A) Seasonal decline of mean daily insect biomass as estimated by independent month specific log-linear regressions (black bars), and our basic mixed effects model with interaction between annual rate of change and a quadratic trend for day number in season. (B), Seasonal phenology of insect biomass (seasonal quantiles of biomass at 5% intervals) across all locations revealing substantial annual variation in peak biomass (solid line) but no direction trend, suggesting no phenological changes have occurred with respect to temporal distribution of insect biomass.

**Fig 4:**
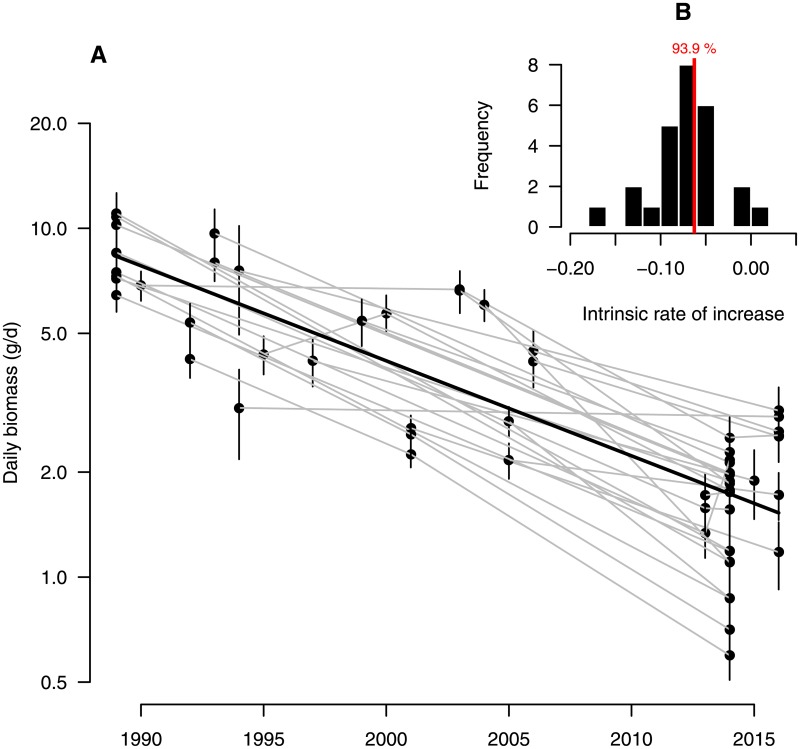
Temporal distribution of insect biomass at selected locations. (A) Daily biomass (mean ±1 se) across 26 locations sampled in multiple years (see S4 Fig for seasonal distributions). (B) Distribution of mean annual rate of decline as estimated based on plot specific log-linear models (annual trend coefficient = −0.053, sd = 0.002, i.e. 5.2% annual decline).

Following corrections for seasonal variation and habitat cluster (basic model, see Materials and methods), the annual trend coefficient of our basic model was significantly negative (annual trend coefficient = −0.063, sd = 0.002, i.e. 6.1% annual decline). Based on this result, we estimate that a major (up to 81.6% [79.7–83.4%]) decline in mid-summer aerial insect biomass has taken place since 1989 (Fig 2A). However, biomass loss was more prominent in mid-summer as compared to the start and end of the season (Fig 3A), indicating that the highest losses occur when biomass is highest during the season (Fig 2B). As such, a seasonally weighted estimate (covering the period 1-April to 30-October; see methods) results in an overall 76.7% [74.8–78.5%] decline over a 27 year period. The pattern of decline is very similar across locations that were sampled more than once (Fig 4), suggesting that the estimated temporal decline based on the entire dataset is not confounded by the sampling procedure. Re-estimation of the annual decline based on 26 locations that have been sampled in more than one year (S4 Fig), revealed a similar rate of decline (76.2%[73.9–78.3%]).

Insect biomass was positively related to temperature and negatively to precipitation (S1 Table). Including lagged effects of weather revealed no effect of either number of frost days, or winter precipitation, on the biomass in the next season (S1 Table). The overall model fit improved as compared to our basic model (R^2^ = 65.4%, Table 3), explaining within and between year variation in insect biomass, but not the overall decline (log(*λ*) = −0.058, sd = 0.002). Over the course of the study period, some temporal changes occurred in the means of the weather variables (S2 Fig), most notably an increase by 0.5°C in mean temperature and a decline 0.2 m/sec in mean wind speed. Yet, these changes either do not have an effect on insect biomass (e.g. wind speed) either are expected to positively affected insect biomass (e.g. increased temperature). Furthermore, a phenological shift with peak biomass earlier in the season could have resulted in lower biomass in the mid-season (Fig 3A), but this does not appear to be the case as none of the seasonal distribution quantiles in biomass showed any temporal trend (Fig 3B).

There was substantial variation in trapped insect biomass between habitat clusters (see Materials and methods), with nutrient-rich grasslands, margins and wasteland containing 43% more insect biomass than nutrient-poor heathland, sandy grassland, and dunes. Yet, the annual rate of decline was similar, suggesting that the decline is not specific to certain habitat types (S5 Fig). To further characterize trap locations, we used past (1989–1994) and present (2012–2015) aerial photographs and quantified land use cover within 200m around the trap locations. On average, cover of arable land decreased, coverage of forests increased, while grassland and surface water did not change much in extent over the last three decades (S3 Fig). Overall, adding land use variables alone did not lead to a substantial improvement of the model fit (R^2^ = 61.3%, Table 3), nor did it affect the annual trend coefficient (log(*λ*) = −0.064, sd = 0.002). While presence of surface water appeared to significantly lower insect biomass, none of the other variables were significantly related to biomass. However, including interactions between the annual trend coefficient and land use variables increased the model fit slightly (Table 3), and revealed significant interactions for all variables except coverage of surface water (S2 Table). These interactions, which were retained in our final model (Table 4), revealed higher rates of decline where coverage of grassland was higher, while lower declines where forest and arable land coverage was higher.

We hypothesized that successional changes in plant community [55] or changes in environmental conditions [9, 18], could have affected the local insect biomass, and hence explain the decline. Plant species inventories that were carried out in the immediate vicinity of the traps and in the same season of trapping, revealed that species richness of trees, shrubs and herbs declined significantly over the course of the study period (S3 Fig). Including species richness in our basic model, i.e. number of tree species and log number of herb species, revealed significant positive and negative effects respectively on insect biomass, but did not affect the annual trend coefficient (S3 Table), explaining some variation between locations rather than the annual trend coefficient. Moreover, and contrary to expectation, trends in herb species richness were weakly negatively correlated with trends in insect biomass, when compared on per plot basis for plots sampled more than once. Ellenberg values of plant species provide a reliable indicator for the environmental conditions such as pH, nitrogen, and moisture [46, 47]. Around trap locations, mean indicators (across all locations) were stable over time, with changes in the order of less than 2% over the course of the study period. Adding these variables to our basic model revealed a significant positive effect of nitrogen and light, and a significant negative effect of Ellenberg temperature on insect biomass, explaining a major part of the variation between the habitat types. However, Ellenberg values did not affect the insect biomass trend coefficient (log(*λ*) = −0.059, sd = 0.003, S3 Table) and only marginally improved the model fit (R^2^ = 61.9%, Table 3). All habitat variables were considered in our final model (Table 4), with the exception of of pH and moisture.

**Fig 5:**
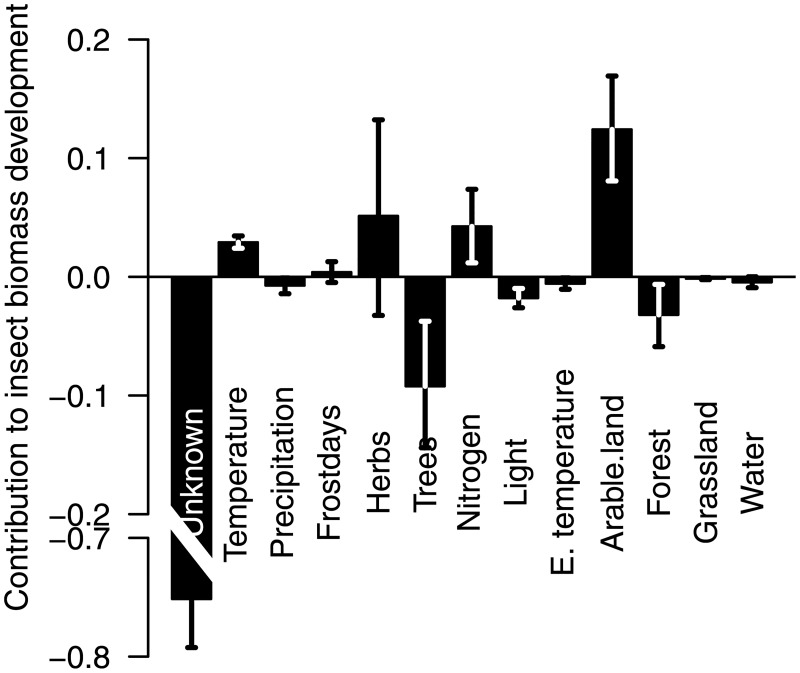
Marginal effects of temporal changes in considered covariates on insect biomass. Each bar represents the rate of change in total insect biomass, as the combined effect of the relevant coefficient (Table 4) and the temporal development of each covariate independently (S2 and S3 Figs).

Our final model, based on including all significant variables from previous models, revealed a higher trend coefficient as compared to our basic model (log(*λ*) = −0.081, sd = 0.006, Table 4), suggesting that temporal developments in the considered explanatory variables counteracted biomass decline to some degree, leading to an even more negative coefficient for the annual trend. The marginal net effect of changes in each covariate over time (see Analysis), showed a positive contribution to biomass growth rate of temporal developments in arable land, herb species richness, and Ellenberg Nitrogen, while negative effects of developments of tree species richness and forest coverage (Fig 5). For example, the negative effect of arable land on biomass (Table 4), in combination with a decrease in coverage of arable land (S3 Fig), have resulted in a net positive effect for biomass (Fig 5). Projections of our final model, while fixing the coefficient for the temporal annual trend log(*λ*) to zero, suggest insect biomass would have remained stable, or even increased by approximately 8% (mean rate = 1.075, 0.849–1.381) over the course of the study period.

## Discussion

Our results document a dramatic decline in average airborne insect biomass of 76% (up to 82% in midsummer) in just 27 years for protected nature areas in Germany. This considerably exceeds the estimated decline of 58% in global abundance of wild vertebrates over a 42-year period to 2012 [56, 57]. Our results demonstrate that recently reported declines in several taxa such as butterflies [7, 25–27, 58], wild bees [8–14] and moths [15–18], are in parallel with a severe loss of total aerial insect biomass, suggesting that it is not only the vulnerable species, but the flying insect community as a whole, that has been decimated over the last few decades. The estimated decline is considerably more severe than the only comparable long term study on flying insect biomass elsewhere [28]. In that study, 12.2m high suction traps were deployed at four locations in the UK over the time period 1973–2002, and showed a biomass decline at one of the four sites only. However, the sampling designs differ considerably between the two studies. Suction traps mainly target high-flying insects, and in that study the catches were largely comprised of flies belonging to the Bibionidae family. Contrary, malaise traps as used in the present study target insects flying close to the ground surface (up to 1 meter), with a much wider diversity of taxa. Future investigations should look into how biomass is distributed among insect species, and how species trends contribute to the biomass decline.

Although the present dataset spans a relatively large number of years (27) and sites (63), the number of repetitions (i.e. multiple years of seasonal distributions at the same locations) was lower (n = 26). We are however confident that our estimated rate of decline in total biomass resembles the true rate of decline, and is not an artifact of site selection. Firstly, our basic model (including an annual rate of decline) outperformed the null-model (without an annual rate of decline; ΔDIC = 822.62 units; Table 3), while at the same time, between-plot variation (i.s. *σ*_*site*_) and residual variation (*v*) decreased by 44.3 and 9.7% respectively, after incorporating an annual rate of decline into the models. Secondly, using only data from sites at which malaise traps were operating in at least two years, we estimated a rate of decline similar to using the full dataset (Fig 4), with the pattern of decline being congruent across locations (S4 Fig). Taken together, there does not seem to be evidence that spatial variation (between sites) in this dataset forms a confounding factor to the estimated temporal trend, and conclude that our estimated biomass decline is representative for lowland protected areas in west Germany.

In light of previously suggested driving mechanisms, our analysis renders two of the prime suspects, i.e. landscape [9, 18, 20] and climate change [15, 18, 21, 37], as unlikely explanatory factors for this major decline in aerial insect biomass in the investigated protected areas. Habitat change was evaluated in terms of changes in plant species composition surrounding the standardized trap locations, and in plant species characteristics (Ellenberg values). Land use changes was evaluated in terms of proportional surface changes in aerial photographs, and not for example changes in management regimes. Given the major decline in insect biomass of about 80%, much stronger relationships would have been expected if changes in habitat and land use were the driving forces, even with the somewhat crude parameters that were at our disposal.

The decline in insect biomass, being evident throughout the growing season, and irrespective of habitat type or landscape configuration, suggests large-scale factors must be involved. While some temporal changes in climatic variables in our study area have taken place, these either were not of influence (e.g. wind speed), or changed in a manner that should have increased insect biomass (e.g temperature). However, we have not exhaustively analysed the full range of climatic variables that could potentially impact insect biomass. For example prolonged droughts, or lack of sunshine especially in low temperatures might have had an effect on insect biomass [59–62]. Agricultural intensification [17, 20] (e.g. pesticide usage, year-round tillage, increased use of fertilizers and frequency of agronomic measures) that we could not incorporate in our analyses, may form a plausible cause. The reserves in which the traps were placed are of limited size in this typical fragmented West-European landscape, and almost all locations (94%) are enclosed by agricultural fields. Part of the explanation could therefore be that the protected areas (serving as insect sources) are affected and drained by the agricultural fields in the broader surroundings (serving as sinks or even as ecological traps) [1, 63–65]. Increased agricultural intensification may have aggravated this reduction in insect abundance in the protected areas over the last few decades. Whatever the causal factors responsible for the decline, they have a far more devastating effect on total insect biomass than has been appreciated previously.

The widespread insect biomass decline is alarming, ever more so as all traps were placed in protected areas that are meant to preserve ecosystem functions and biodiversity. While the gradual decline of rare insect species has been known for quite some time (e.g. specialized butterflies [9, 66]), our results illustrate an ongoing and rapid decline in total amount of airborne insects active in space and time. Agricultural intensification, including the disappearance of field margins and new crop protection methods has been associated with an overall decline of biodiversity in plants, insects, birds and other species in the current landscape [20, 27, 67]. The major and hitherto unrecognized loss of insect biomass that we report here for protected areas, adds a new dimension to this discussion, because it must have cascading effects across trophic levels and numerous other ecosystem effects. There is an urgent need to uncover the causes of this decline, its geographical extent, and to understand the ramifications of the decline for ecosystems and ecosystem services.

## Supporting information

S1 Appendix Malaise trap permissions. (PDF)

S2 Appendix Malaise traps. (PDF)

S1 Code (PDF)

S1 Dataset (CSV)

S2 Dataset (CSV)

S1 Fig Map of study area. Insect trap locations (yellow points) in Nordrhein-Westfalen (n = 57), Rheinland-Pfalz (n = 1) and Brandenburg (n = 5), as well as weather stations (crosses) used in the present analysis.
(TIFF)

S2 Fig Temporal variation in weather variables. Annual means (A-C), daily means (D-F), and mean daily residual values (G-I) of temperature, precipitation and wind speed respectively. In all panels, black lines depict data while blue and red lines represent long term and seasonal fitted means of the variables, respectively.
(PDF)

S3 Fig Land use and plant species richness changes. Mean land use in 1989–1994 (A) and 2012–2014 (B), based on aerial photograph analysis at 63 protected areas show a decrease of arable land and an increase in forested area over the past 25 years. (C) Changes in plants species richness for herbs (black) shrubs (red) and trees (blue). Annual means as well as mean trends are depicted in the corresponding colors. Linear trends are based on generalized linear mixed effects models with a Poisson error distribution and a random intercept effect for location. Note, zero values for tree and shrub species not depicted.
(PDF)

S4 Fig Seasonal profiles of daily biomass across 26 locations. For each location, different colors represent different years, with time color-coded from green (1989) to red (2016). X-axis represents day number (January 1 = 0).
(PDF)

S5 Fig Daily biomass of insects over time for two habitat clusters. Boxplots depict the distribution of insect biomass pooled over all traps and catches in each year at trap locations in nutrient-poor heathland, sandy grassland, and dunes (A), and in nutrient-rich grasslands, margins and wasteland (B). Grey lines depict the fitted mean (+95% posterior credible intervals), while the black lines the mean estimated trend. Estimated annual decline amounts to 7.5%(6.6–8.4) for habitat cluster 1, as compared to 5.2% (4.8–5.5) habitat cluster 2. Models fitted independently for each habitat location. Color gradient in all panels range from 1989 (blue) to 2016 (orange).
(PDF)

S1 Table Posterior parameter estimates of the mixed effects model including weather variables. For each included variable, the corresponding coefficient posterior mean, standard deviation and 95% credible intervals are given. P-values are calculated empirically based on posterior distributions of coefficients.
(PDF)

S2 Table Posterior parameter estimates of the mixed effects model including land use variables and interactions. For each included variable, the corresponding coefficient posterior mean, standard deviation and 95% credible intervals are given. P-values are calculated empirically based on posterior distributions of coefficients.
(PDF)

S3 Table Posterior parameter estimates of the mixed effects model including habitat variables. For each included variable, the corresponding coefficient posterior mean, standard deviation and 95% credible intervals are given. P-values are calculated empirically based on posterior distributions of coefficients.
(PDF)
