# Supplementary material for: More than 75 percent decline over 27 years in total flying insect biomass in protected areas
Source: PLoS One. 2017 Oct 18;12(10):e0185809. doi: 10.1371/journal.pone.0185809 (PMC5646769; doi:10.1371/journal.pone.0185809)
Supplement: S1 Appendix — (PDF) [file pone.0185809.s001.pdf]

S1 Appendix. Malaise trap permissions According to the German laws and regulations, permissions are necessary in order to perform investigations with malaise traps, at all locations. Permissions for investigations outside as well as within protected areas for the investigations were given by the following authorities (Höheren & Unteren Landschaftsbehörden). These permits include cover both the entry into protected areas, as well as the trapping of species that have a protected status pursuant to German law (Bundesartenschutzverordnung (BArtSchV)) and Bundesnaturschutzgesetz (BNatSchG)).

Authorities issuing the permissions for the investigations are listed below (location abbreviations in brackets).

- Struktur und Genehmigungsdirektion Nord (SGD), Rheinland-Pfalz (POM1)
- Landesamt für Umwelt (LfU), Brandenburg, Biologische Station Beeskow (BKL1, GRI1, LAN1)
- Untere Landschaftsbehörde, Kreis Kleve (SCH1)
- Untere Landschaftsbehörde, Kreis Viersen (BRA1-4, RAH1-2)
- Untere Landschaftsbehörde, Kreis Wesel (BIR1, BIS1-10, HUK1, KAN1-2, LOO1, PLI1-2, SLL1, XAN1-2)
- Untere Landschaftsbehörde, Kreis Mettmann (PIM1)
- Untere Landschaftsbehörde,Kreis Düren (SOL1-2)
- Untere Landschaftsbehörde, Stadt D"usseldorf (URD1-2)
- Untere Landschaftsbehörde, Stadt Köln (WAN1)
- Untere Landschaftsbehörde, Stadt Krefeld (BOO1, CAR1, GEO1, NIE1, ORB1-2, SPE1)
- Untere Landschaftsbehörde, Oberbergischer Kreis (LIN1-2)
- Untere Landschaftsbehörde, Rhein Kreis Neuss (SPE2)
- Untere Landschaftsbehörde, Rhein-Sieg-Kreis (WAH1-6, WAN3-4)
