# Supplementary material for: More than 75 percent decline over 27 years in total flying insect biomass in protected areas
Source: PLoS One. 2017 Oct 18;12(10):e0185809. doi: 10.1371/journal.pone.0185809 (PMC5646769; doi:10.1371/journal.pone.0185809)
Supplement: S2 Appendix — (PDF) [file pone.0185809.s002.pdf]

S2 Appendix. Malaise traps. In this appendix we give more details about the malaise traps, collecting design, and accompanying methods of biomass measurement as designed and applied by the Entomological Society Krefeld. These are also described in German publications [29-31].

The traps used for our research were identically built by the Entomological Society Krefeld itself. Since 1982 the Entomological Society Krefeld has produced malaise traps on the basis of a single cut pattern. This cut pattern is preserved in the archive of the Entomological Society Krefeld and has served as a template for the construction of all traps used in this research. Likewise, the connections of the trap with the bottle in which insects were collected were always constructed in exactly the same way based on reproductions of an template produced in 1982. These self-constructed and identical traps were very similar to the bi-coloured Malaise traps first described by Townes (1972) [32]. All aspects of the sampling was therefore standardized: trap construction, size and design (see figures below), colour, netting and stainless steel connections with the collection bottles.

The traps were also applied using a fixed sampling design. Each trap was placed in such a way that there was no shadow on the roof of the trap in order not to influence the sampling. The catch head was aligned to the south by using of a compass to make sure that the entry of the insects into the traps was always from the east and west. At the four corners, wooden poles were placed to adjust the height of the trap and thus the active catch area to the set standard. In all cases, the trap was tightly connected to the ground to ensure that no insects could slip between the ground and the netting.

In summary, the standardization of the sampling design were undertaken with the idea of quantitative analyses of flying insect biomass across years and sites in protected areas.

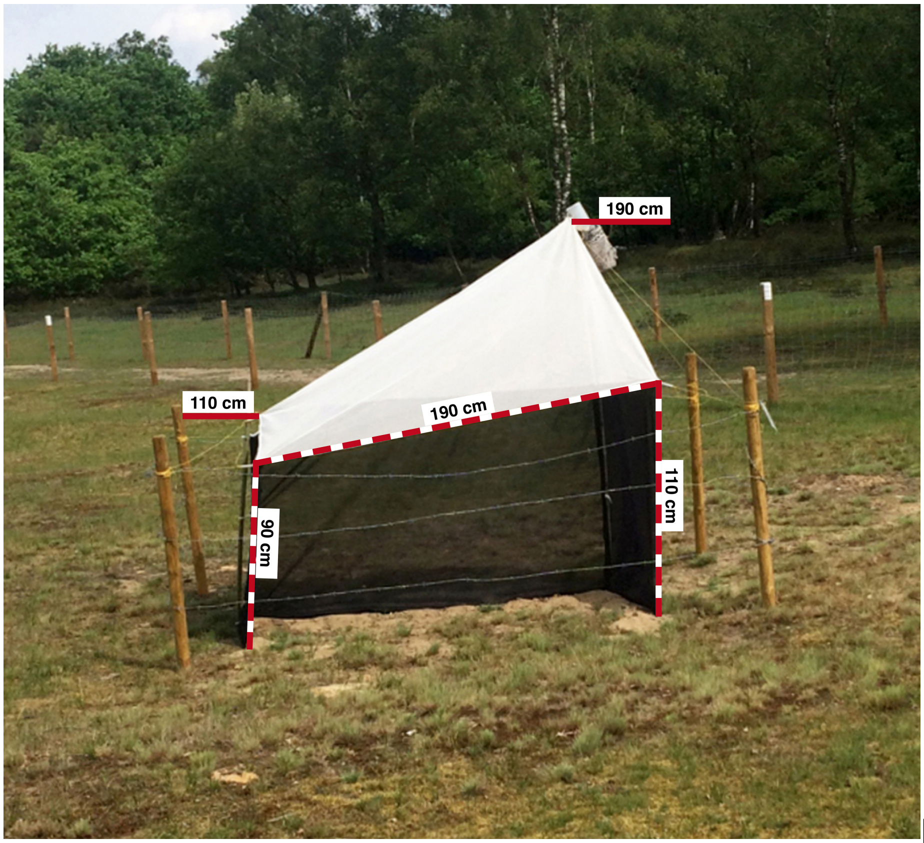

[!h]

S2 Appendix, Fig. 1. Malaise trap design with fixed dimension, materials and orientation. Middle height from the ground at the catch head, directed to the south: 190cm. Corner height from ground of the opening on the south side: 110cm. Corner height from ground of the opening on the north side: 90cm. Length of the opening - 190cm. Catch area: 1.89 m^2 west and 1.89 m^2 east: total catch area (opening) 3.78 m^2.

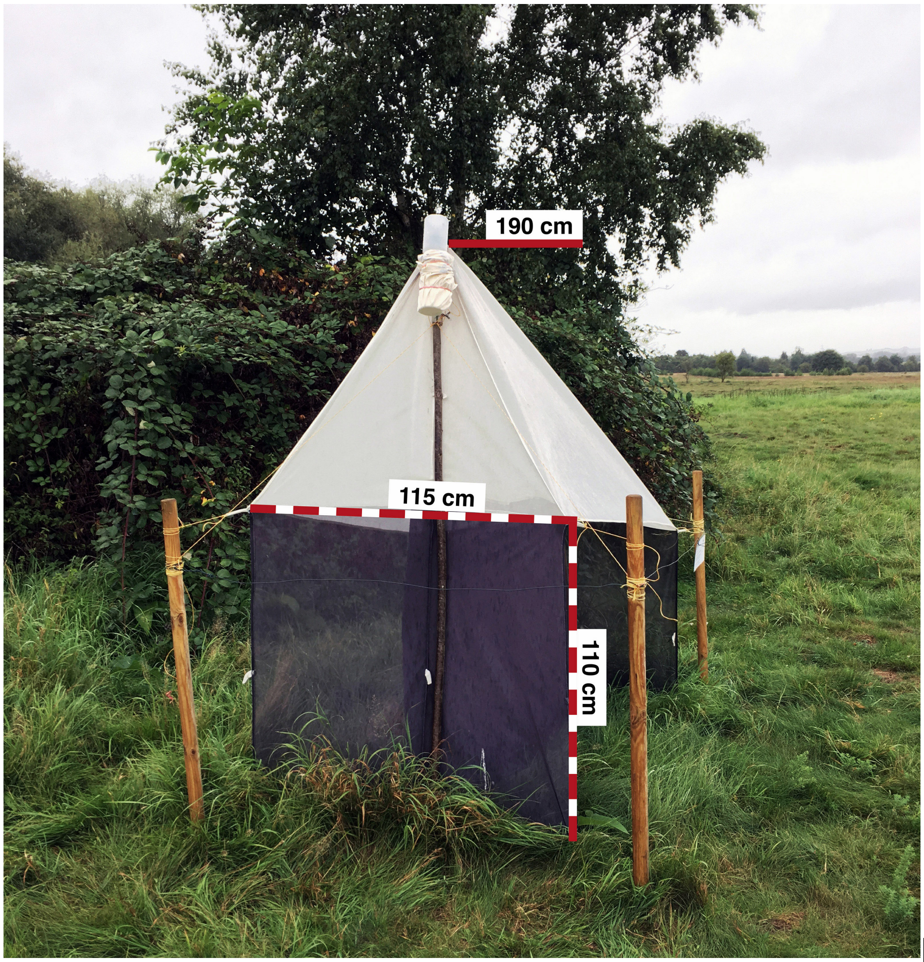

[!h]

S2 Appendix, Fig. 2. Malaise trap design with fixed dimension, materials and orientation
