# Supplementary material for: More than 75 percent decline over 27 years in total flying insect biomass in protected areas
Source: PLoS One. 2017 Oct 18;12(10):e0185809. doi: 10.1371/journal.pone.0185809 (PMC5646769; doi:10.1371/journal.pone.0185809)
Supplement: S1 Code — (PDF) [file pone.0185809.s003.pdf]

S1 Code

In this supporting information we provide the R-code and JAGS-code used to run the models in the main text. R-packages on which the analysis depends, are included at the start. The code used two pieces of data as provided in S1 Dataset and S2 dataset, being the biomass data and covariate data respectively.

```
# required libraries
library(R2jags)
# Function to standardize variables
standardize <- function(x,mu=mean(x),sig=sd(x)) (x-mu)/sig
# read the data
data <- read.table("S1_Dataset.csv",header=TRUE,sep=",")
model.frame<- read.table("S2_Dataset.csv",header=TRUE,sep=",")
summary(data)
summary(model.frame)
```

Below, for each of the seven models in Table 3 in the main text, we first create a dataset based on the two loaded datasets, write a JAGS model file, and run the model through JAGS.

Null model

Setup the data for running the JAGS model:

```
jagsdataNull<-list(
        m = data$biomass,
        index1= with(model.frame,tapply(1:nrow(model.frame),potID,min))[data$potID],
        index2=with(model.frame,tapply(1:nrow(model.frame),potID,max))[data$potID],
        plot=    as.numeric(model.frame$plot),
        loctype = as.numeric(data$location.type[match(model.frame$potID,data$potID)]),
        daynr=   as.numeric((model.frame$daynr-mean(data$mean.daynr))/ sd(data$mean.daynr)),
        daynr2=  as.numeric((model.frame$daynr-mean(data$mean.daynr))/ sd(data$mean.daynr))^2,
        ndaily= nrow(model.frame),
        n=nrow(data),
        nrandom=max( as.numeric(model.frame$plot))
)
```

Write JAGS model file:

```
sink("NullModel.jag")
cat("
model{
for( i in 1:n){
m[i] ~ dnorm(sum(y[index1[i]:index2[i]]),tau[i])
tau[i] <- 1/Var[i]
Var[i] <- sum( vr[index1[i]:index2[i]] )
}
for(i in 1:ndaily){
y[i] <- exp(z[i])
z[i] <- int +
c[1]*daynr[i] + c[2]*daynr2[i]+
b[loctype[i]] +
eps[plot[i]]
vr[i]<- exp( 2* z[i]+ lvar ) * (exp(lvar)-1 )
}
int~  dnorm(0,.01)
b[1]<-0
for( i in 2:3) { b[i] ~ dnorm(0,.01)  }
for( i in 1:2) { c[i] ~ dnorm(0,.01)  }
sdhat ~ dunif(0,5)
lvar <- pow(sdhat,2)
for(i in 1:nrandom){
eps[i] ~ dnorm(0,tau.re)
}
tau.re<- pow(sd.re,-2)
sd.re ~ dunif(0,1)
}
")
sink(NULL)
```

Run the model:

```
# Model NULL
parametersNull=c("int","b","c","eps","sdhat","sd.re")
jagsmod0<- jags(jagsdataNull,inits=NULL,parametersNull,
        "NullModel.jag",n.chains=3,n.iter=12000,n.burnin=2000,n.thin=10)
jagsmod0
```

Basic model

Setup the data for running the JAGS model:

```
# prepare a list with data elements for JAGS
jagsdataBasic<-list(
        m = data$biomass,
        index1= with(model.frame,tapply(1:nrow(model.frame),potID,min))[data$potID],
        index2=with(model.frame,tapply(1:nrow(model.frame),potID,max))[data$potID],
        plot=    as.numeric(model.frame$plot),
        loctype = as.numeric(data$location.type[match(model.frame$potID,data$potID)]),
        daynr=   as.numeric((model.frame$daynr-mean(data$mean.daynr))/ sd(data$mean.daynr)),
        daynr2=  as.numeric((model.frame$daynr-mean(data$mean.daynr))/ sd(data$mean.daynr))^2,
        year=model.frame$year-1988,
        ndaily= nrow(model.frame),
        n=nrow(data),
        nrandom=max( as.numeric(model.frame$plot))
)
```

Write JAGS model file:

```
sink("BasicModel.jag")
cat("
model{
for( i in 1:n){
m[i] ~ dnorm(sum(y[index1[i]:index2[i]]),tau[i])
tau[i] <- 1/Var[i]
Var[i] <- sum( vr[index1[i]:index2[i]] )
}
for(i in 1:ndaily){
y[i] <- exp(z[i])
z[i] <- int + log.lambda*year[i]  +
c[1]*daynr[i] + c[2]*daynr2[i]+
c[3]*daynr[i]*year[i]+c[4]*daynr2[i]*year[i]+
b[loctype[i]] + eps[plot[i]]
vr[i]<- exp( 2* z[i]+ lvar ) * (exp(lvar)-1 )
}
int~  dnorm(0,.01)
log.lambda~  dnorm(0,.01)
b[1]<-0
for( i in 2:3) { b[i] ~ dnorm(0,.01)  }
for( i in 1:4) { c[i] ~ dnorm(0,.01)  }
sdhat ~ dunif(0,5)
lvar <- pow(sdhat,2)
for(i in 1:nrandom){
eps[i] ~ dnorm(0,tau.re)
}
tau.re<- pow(sd.re,-2)
sd.re ~ dunif(0,1)
}
")
sink(NULL)
```

Run the model:

```
parametersBasic=c("int","log.lambda","b","c","eps","sdhat","sd.re")
jagsmodBasic<- jags(jagsdataBasic,inits=NULL,parameters=parametersBasic,
        "BasicModel.jag",       n.chains=3,n.iter=24000,n.burnin=4000,n.thin=10)
```

Weather model

Setup the data for running the JAGS model:

```
jagsdataWeather<-list(
        m = data$biomass,
        index1= with(model.frame,tapply(1:nrow(model.frame),potID,min))[data$potID],
        index2=with(model.frame,tapply(1:nrow(model.frame),potID,max))[data$potID],
        plot=    as.numeric(model.frame$plot),
        loctype = as.numeric(data$location.type[match(model.frame$potID,data$potID)]),
        daynr=   as.numeric((model.frame$daynr-mean(data$mean.daynr))/ sd(data$mean.daynr)),
        daynr2=  as.numeric((model.frame$daynr-mean(data$mean.daynr))/ sd(data$mean.daynr))^2,
        year=model.frame$year-1988,
        ndaily= nrow(model.frame),
        n=nrow(data),
        nrandom=max( as.numeric(model.frame$plot)),
        temp=standardize(model.frame$temperature),
        prec=standardize(model.frame$precipitation),
        wind=standardize(model.frame$wind.speed),
        frost=standardize(model.frame$frostdays),
        w.prec=standardize(model.frame$sum.precW)
)
```

Write JAGS model file:

```
sink("WeatherModel.jag")
cat('
model{
for( i in 1:n){
m[i] ~ dnorm(sum(y[index1[i]:index2[i]]),tau[i])
tau[i] <- 1/Var[i]
Var[i] <- sum( vr[index1[i]:index2[i]] )
}
for(i in 1:ndaily){
y[i] <- exp(z[i])
z[i] <- int +
log.lambda*year[i]  +
c[1]*daynr[i] + c[2]*daynr2[i]+
c[3]*daynr[i]*year[i] + c[4]*daynr2[i]*year[i]+
w[1]*temp[i] + w[2]*prec[i] + w[3]*wind[i]  +
w[4]*frost[i]+ w[5]*w.prec[i]+
b[loctype[i]] +
eps[plot[i]]
vr[i]<- exp( 2* z[i]+ lvar ) * (exp(lvar)-1 )
}
int~  dnorm(0,.01)
log.lambda~  dnorm(0,.01)
b[1]<-0
for( i in 2:3) { b[i] ~ dnorm(0,.01)  }
for( i in 1:4) { c[i] ~ dnorm(0,.01)  }
for( i in 1:5) { w[i] ~ dnorm(0,.01)  }
sdhat ~ dunif(0,5
lvar <- pow(sdhat,2)
for(i in 1:nrandom){
eps[i] ~ dnorm(0,tau.re)
}
tau.re<- pow(sd.re,-2)
sd.re ~ dunif(0,1)
}
')
sink(NULL)
```

Run the model:

```
parametersWeather=c("int","log.lambda","b","c","w","eps","sdhat","sd.re")
jagsmodWeather<- jags(jagsdataWeather,NULL,
                parametersWeather,"WeatherModel.jag",
                n.chains=3,n.iter=24000,n.burnin=4000,n.thin=10)
jagsmodWeather
```

Habitat model

Setup the data for running the JAGS model:

```
jagsdataHabitat<-list(
        m = data$biomass,
        index1= with(model.frame,tapply(1:nrow(model.frame),potID,min))[data$potID],
        index2=with(model.frame,tapply(1:nrow(model.frame),potID,max))[data$potID],
        plot=    as.numeric(model.frame$plot),
        loctype = as.numeric(data$location.type[match(model.frame$potID,data$potID)]),
        daynr=   as.numeric((model.frame$daynr-mean(data$mean.daynr))/ sd(data$mean.daynr)),
        daynr2=  as.numeric((model.frame$daynr-mean(data$mean.daynr))/ sd(data$mean.daynr))^2,
        year=model.frame$year-1988,
        ndaily= nrow(model.frame),
        n=nrow(data),
        nrandom=max( as.numeric(model.frame$plot)),
        herbs=log(model.frame$nHerbs),
        Trees=standardize(model.frame$nTrees),
        nitro=standardize(model.frame$Nitrogen),
        pH=standardize(model.frame$pH),
        moist=standardize(model.frame$Moisture),
        light=standardize(model.frame$Light),
        etemp=standardize(model.frame$ellenTemperature)
)
```

Write JAGS model file:

```
sink("HabitatModel.jag")
cat("
model{
for( i in 1:n){
m[i] ~ dnorm(sum(y[index1[i]:index2[i]]),tau[i])
tau[i] <- 1/Var[i]
Var[i] <- sum( vr[index1[i]:index2[i]] )
}
for(i in 1:ndaily){
y[i] <- exp(z[i])
z[i] <- int +
log.lambda*year[i]  +
c[1]*daynr[i] + c[2]*daynr2[i]+
c[3]*daynr[i]*year[i]+c[4]*daynr2[i]*year[i]+
d[1]*herbs[i]+d[2]*Trees[i]+
d[3]*nitro[i]+d[4]*pH[i]+d[5]*moist[i]+
d[6]*light[i]+d[7]*etemp[i]+
b[loctype[i]] +
eps[plot[i]]
vr[i]<- exp( 2* z[i]+ lvar ) * (exp(lvar)-1 )
}
int~  dnorm(0,.01)
log.lambda~  dnorm(0,.01)
b[1]<-0
for( i in 2:3) { b[i] ~ dnorm(0,.01)  }
for( i in 1:4) { c[i] ~ dnorm(0,.01)  }
for( i in 1:7) { d[i] ~ dnorm(0,.01)  }
sdhat ~ dunif(0,5)
lvar <- pow(sdhat,2)
for(i in 1:nrandom){
eps[i] ~ dnorm(0,tau.re)
}
tau.re<- pow(sd.re,-2)
sd.re ~ dunif(0,1)
}
")
sink(NULL)
```

Run the model:

```
parametersHabitat=c("int","log.lambda","b","c","d","eps","sdhat","sd.re")
jagsmodHabitat<- jags(jagsdataHabitat,NULL,parametersHabitat,
                "HabitatModel.jag",n.chains=3,n.iter=24000,n.burnin=4000,n.thin=10)
jagsmodHabitat
```

Landuse model

Setup the data for running the JAGS model:

```
jagsdataLandUse<-list(
        m = data$biomass,
        index1= with(model.frame,tapply(1:nrow(model.frame),potID,min))[data$potID],
        index2=with(model.frame,tapply(1:nrow(model.frame),potID,max))[data$potID],
        plot=    as.numeric(model.frame$plot),
        loctype = as.numeric(data$location.type[match(model.frame$potID,data$potID)]),
        daynr=   as.numeric((model.frame$daynr-mean(data$mean.daynr))/ sd(data$mean.daynr)),
        daynr2=  as.numeric((model.frame$daynr-mean(data$mean.daynr))/ sd(data$mean.daynr))^2,
        year=model.frame$year-1988,
        ndaily= nrow(model.frame),
        n=nrow(data),
        nrandom=max( as.numeric(model.frame$plot)),
        agribuf=(model.frame$Arable.land),
        forebuf=(model.frame$Forest),
        grasbuf=(model.frame$Grassland),
        water=(model.frame$Water)
        )
```

```
sink("LandUseModel.jag")
cat("
model{
for( i in 1:n){
m[i] ~ dnorm(sum(y[index1[i]:index2[i]]),tau[i])
tau[i] <- 1/Var[i]
Var[i] <- sum( vr[index1[i]:index2[i]] )
}
for(i in 1:ndaily){
y[i] <- exp(z[i])
z[i] <- int +
log.lambda*year[i]  +
c[1]*daynr[i] + c[2]*daynr2[i]+
c[3]*daynr[i]*year[i] + c[4]*daynr2[i]*year[i] +
d[1]*agribuf[i]+d[2]*forebuf[i]+
d[3]*grasbuf[i]+d[4]*water[i]+
b[loctype[i]]+  eps[plot[i]]
vr[i]<- exp( 2* z[i]+ lvar ) * (exp(lvar)-1 )
}
int~  dnorm(0,.01)
log.lambda~  dnorm(0,.01)
b[1]<-0
for( i in 2:3) { b[i] ~ dnorm(0,.01)  }
for( i in 1:4) { c[i] ~ dnorm(0,.01)  }
for( i in 1:4) { d[i] ~ dnorm(0,.01)  }
sdhat ~ dunif(0,5)
lvar <- pow(sdhat,2)
for(i in 1:nrandom){
eps[i] ~ dnorm(0,tau.re)
}
tau.re<- pow(sd.re,-2)
sd.re ~ dunif(0,1)
}
")
sink(NULL)
```

Run the model:

```
parametersLandUse=c("int","log.lambda","b","c","d","eps","sdhat","sd.re")
jagsmodLandUse<- jags(LandUseModel,NULL,parametersLandUse,"LandUseModel.jag",
        n.chains=3,n.iter=24000,n.burnin=4000,n.thin=10)
jagsmodLandUse
```

Landuse with interactions model

Setup the data for running the JAGS model:

```
jagsdataLandUse<-list(
        m = data$biomass,
        index1= with(model.frame,tapply(1:nrow(model.frame),potID,min))[data$potID],
        index2=with(model.frame,tapply(1:nrow(model.frame),potID,max))[data$potID],
        plot=    as.numeric(model.frame$plot),
        loctype = as.numeric(data$location.type[match(model.frame$potID,data$potID)]),
        daynr=   as.numeric((model.frame$daynr-mean(data$mean.daynr))/ sd(data$mean.daynr)),
        daynr2=  as.numeric((model.frame$daynr-mean(data$mean.daynr))/ sd(data$mean.daynr))^2,
        year=model.frame$year-1988,
        ndaily= nrow(model.frame),
        n=nrow(data),
        nrandom=max( as.numeric(model.frame$plot)),
        agribuf=(model.frame$Arable.land),
        forebuf=(model.frame$Forest),
        grasbuf=(model.frame$Grassland),
        water=(model.frame$Water)
        )
```

Write JAGS model file:

```
sink("LandUseIntModel.jag")
cat("
model{
for( i in 1:n){
m[i] ~ dnorm(sum(y[index1[i]:index2[i]]),tau[i])
tau[i] <- 1/Var[i]
Var[i] <- sum( vr[index1[i]:index2[i]] )
}
for(i in 1:ndaily){
y[i] <- exp(z[i])
z[i] <- int +
log.lambda*year[i]  +
c[1]*daynr[i] + c[2]*daynr2[i]+
c[3]*daynr[i]*year[i] + c[4]*daynr2[i]*year[i] +
d[1]*agribuf[i]+d[2]*forebuf[i]+
d[3]*grasbuf[i]+d[4]*water[i]+
d[5]*agribuf[i]*year[i]+d[6]*forebuf[i]*year[i]+
d[7]*grasbuf[i]*year[i]+d[8]*water[i]*year[i]+
b[loctype[i]] +
eps[plot[i]]
vr[i]<- exp( 2* z[i]+ lvar ) * (exp(lvar)-1 )
}
int~  dnorm(0,.01)
log.lambda~  dnorm(0,.01)
b[1]<-0
for( i in 2:3) { b[i] ~ dnorm(0,.01)  }
for( i in 1:4) { c[i] ~ dnorm(0,.01)  }
for( i in 1:8) { d[i] ~ dnorm(0,.01)  }
sdhat ~ dunif(0,5)
lvar <- pow(sdhat,2)
for(i in 1:nrandom){
eps[i] ~ dnorm(0,tau.re)
}
tau.re<- pow(sd.re,-2)
sd.re ~ dunif(0,1)
}
")
sink(NULL)
```

Run the model:

```
parametersLanduse=c("int","log.lambda","b","c","d","eps","sdhat","sd.re")
LandUseIntModel<- jags(LandUseData,NULL,parametersLanduse,"LandUseIntModel.jag",
        n.chains=3,n.iter=24000,n.burnin=4000,n.thin=10)
LandUseIntModel
```

Full model

Setup the data for running the JAGS model:

```
jagsdataFull<-list(
        m = data$biomass,
        index1= with(model.frame,tapply(1:nrow(model.frame),potID,min))[data$potID],
        index2=with(model.frame,tapply(1:nrow(model.frame),potID,max))[data$potID],
        plot=    as.numeric(model.frame$plot),
        loctype = as.numeric(data$location.type[match(model.frame$potID,data$potID)]),
        daynr=   as.numeric((model.frame$daynr-mean(data$mean.daynr))/ sd(data$mean.daynr)),
        daynr2=  as.numeric((model.frame$daynr-mean(data$mean.daynr))/ sd(data$mean.daynr))^2,
        year=model.frame$year-1988,
        ndaily= nrow(model.frame),
        n=nrow(data),
        nrandom=max( as.numeric(model.frame$plot)),
        temp=standardize(model.frame$temperature),
        prec=standardize(model.frame$precipitation),
        frost=standardize(model.frame$frostdays),
        herbs=log(model.frame$nHerbs),
        Trees=standardize(model.frame$nTrees),
        nitro=standardize(model.frame$Nitrogen),
        light=standardize(model.frame$Light),
        etemp=standardize(model.frame$ellenTemperature),
        agribuf=(model.frame$Arable.land),
        forebuf=(model.frame$Forest),
        grasbuf=(model.frame$Grassland),
        water=(model.frame$Water)
        )
```

Write JAGS model file:

```
sink("FullModel.jag")
cat(
model{
for(i in 1:n){
m[i]~dnorm(sum(y[index1[i]:index2[i]]),tau[i])
tau[i]<-1/Var[i]
Var[i]<-sum(vr[index1[i]:index2[i]])
}
for(i in 1:ndaily){
y[i]<-exp(z[i])
z[i]<-int+log.lambda*year[i]+
c[1]*daynr[i]+c[2]*daynr2[i]+
c[3]*daynr[i]*year[i]+c[4]*daynr2[i]*year[i]+
w[1]*temp[i]+w[2]*prec[i]+
w[3]*frost[i]+ b[loctype[i]]+
d[1]*herbs[i]+d[2]*Trees[i]+
d[3]*nitro[i]+d[4]*light[i]+d[5]*etemp[i]+
d[6]*agribuf[i]+d[7]*forebuf[i]+d[8]*grasbuf[i]+d[9]*water[i]+
d[10]*agribuf[i]*year[i]+d[11]*forebuf[i]*year[i]+d[12]*grasbuf[i]*year[i]+
eps[plot[i]]
vr[i]<-exp(2*z[i]+lvar)*(exp(lvar)-1)
}
int~dnorm(0,.01)
log.lambda~ dnorm(0,.01)
b[1]<-0
for(i in 2:3){b[i]~dnorm(0,.01)}
for(i in 1:4){c[i]~ dnorm(0,.01)}
for(i in 1:3){w[i]~dnorm(0,.01)}
for(i in 1:12){d[i]~dnorm(0,.01)}
sdhat~dunif(0,5)
lvar<-pow(sdhat,2)
for(i in 1:nrandom){
```

```
eps[i]~dnorm(0,tau.re)
}
tau.re<-pow(sd.re,-2)
sd.re~dunif(0,1)
}
")
sink(NULL)
```

Run the model:

```
parametersFull<- c("int","log.lambda","b","c","w","d","eps","sdhat","sd.re")
FullModel<- jags(jagsdataFull,NULL,parametersFull,"FullModel.jag",
                n.chains=3,n.iter=24000,n.burnin=       4000,n.thin=10)
FullModel
```
