# Supplementary figures and images for: More than 75 percent decline over 27 years in total flying insect biomass in protected areas
Source: PLoS One. 2017 Oct 18;12(10):e0185809. doi: 10.1371/journal.pone.0185809 (PMC5646769; doi:10.1371/journal.pone.0185809)

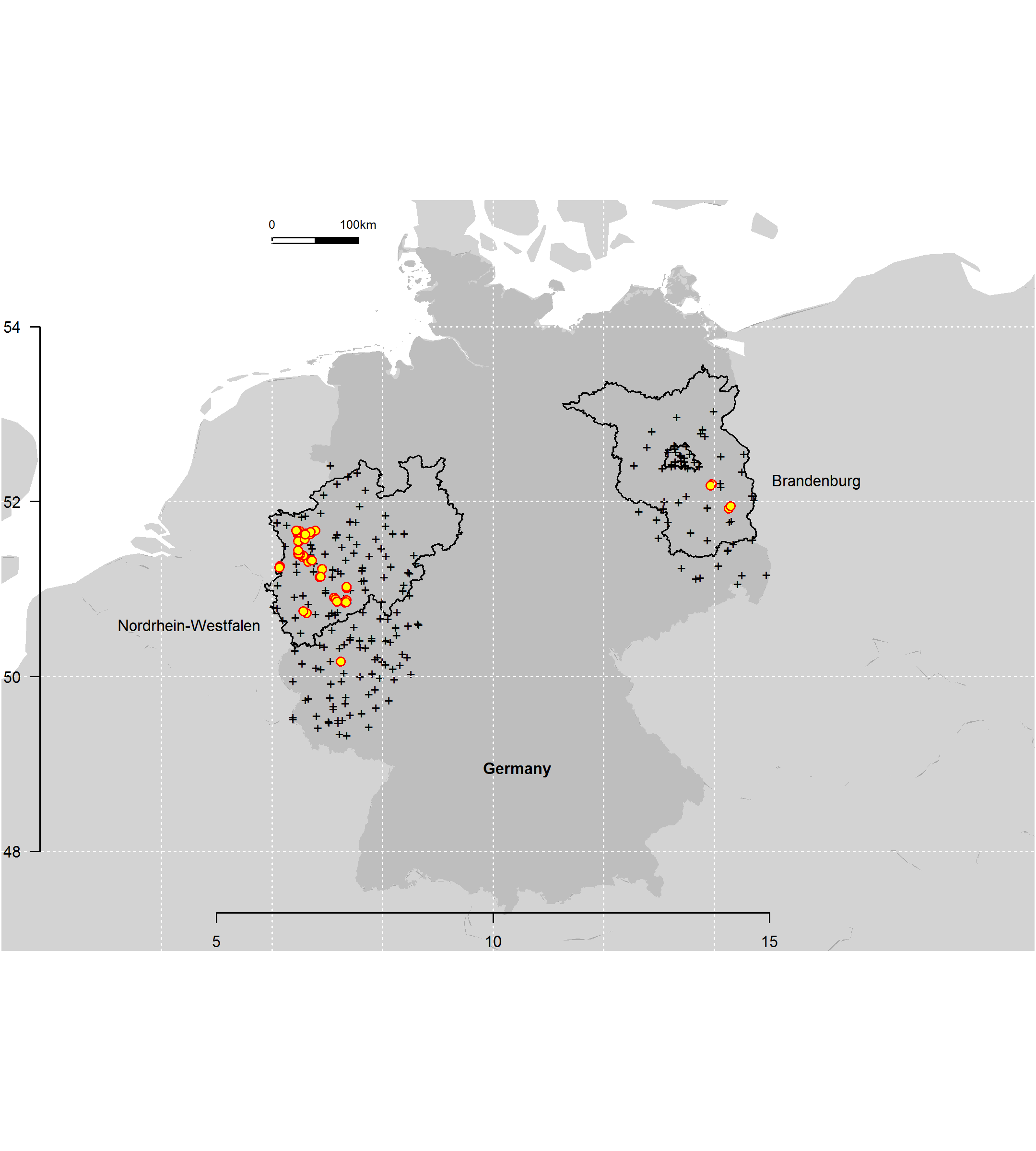

Supplement: S1 Fig — Insect trap locations (yellow points) in Nordrhein-Westfalen (n = 57), Rheinland-Pfalz (n = 1) and Brandenburg (n = 5), as well as weather stations (crosses) used in the present analysis. (TIFF) [file pone.0185809.s006.tiff]

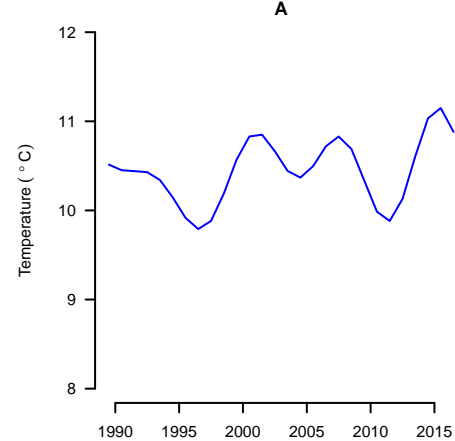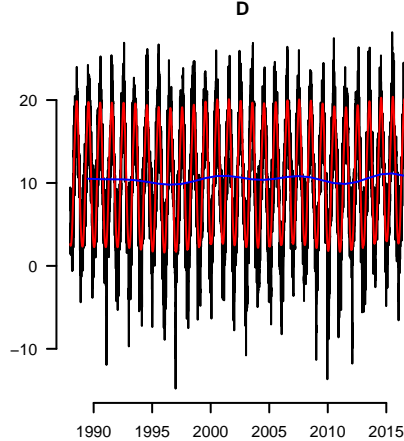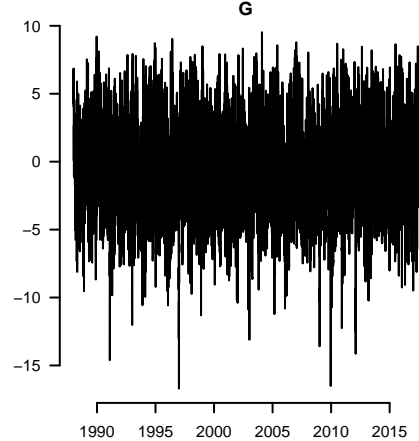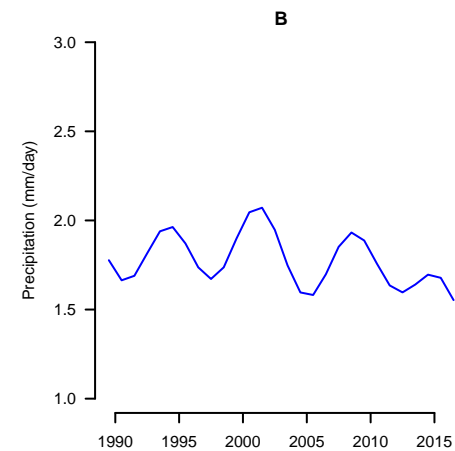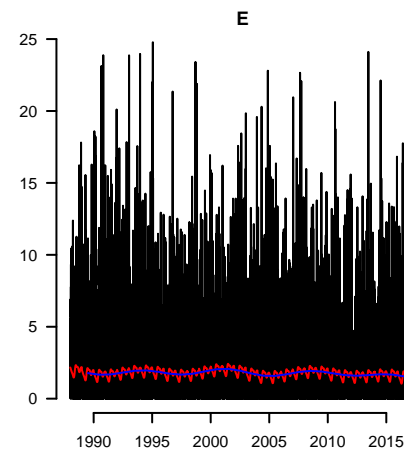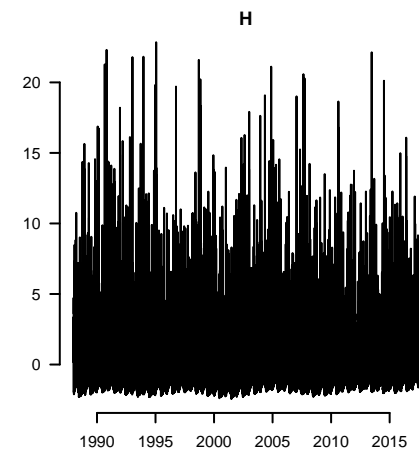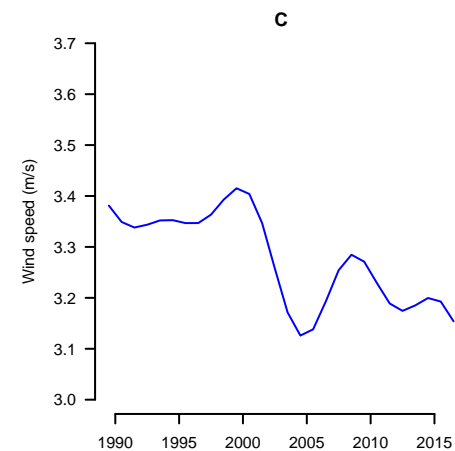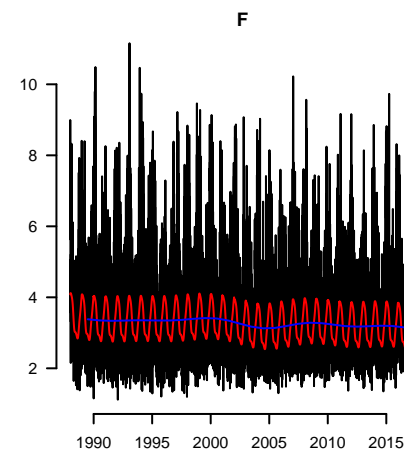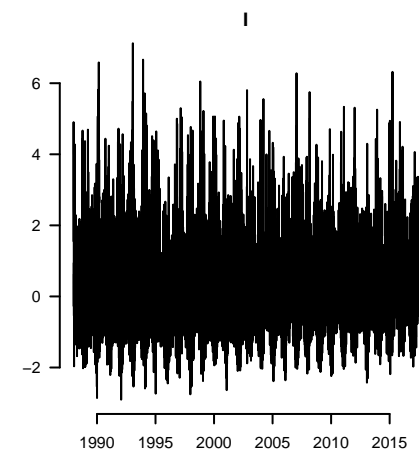

Supplement: S2 Fig — Annual means (A-C), daily means (D-F), and mean daily residual values (G-I) of temperature, precipitation and wind speed respectively. In all panels, black lines depict data while blue and red lines represent long term and seasonal fitted means of the variables, respectively. (PDF) [file pone.0185809.s007.pdf]

**A**  
1989–1994

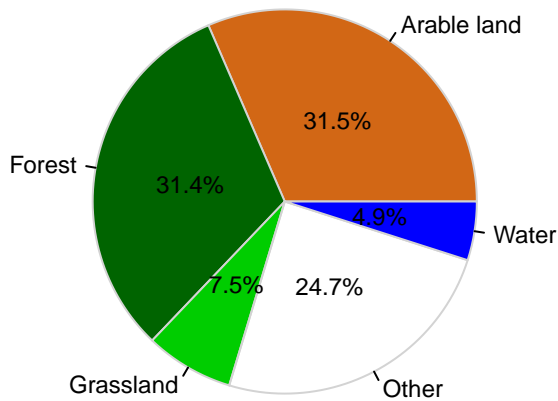

**B**  
2012–2014

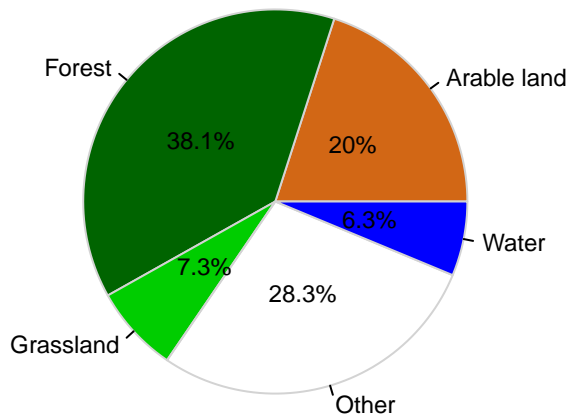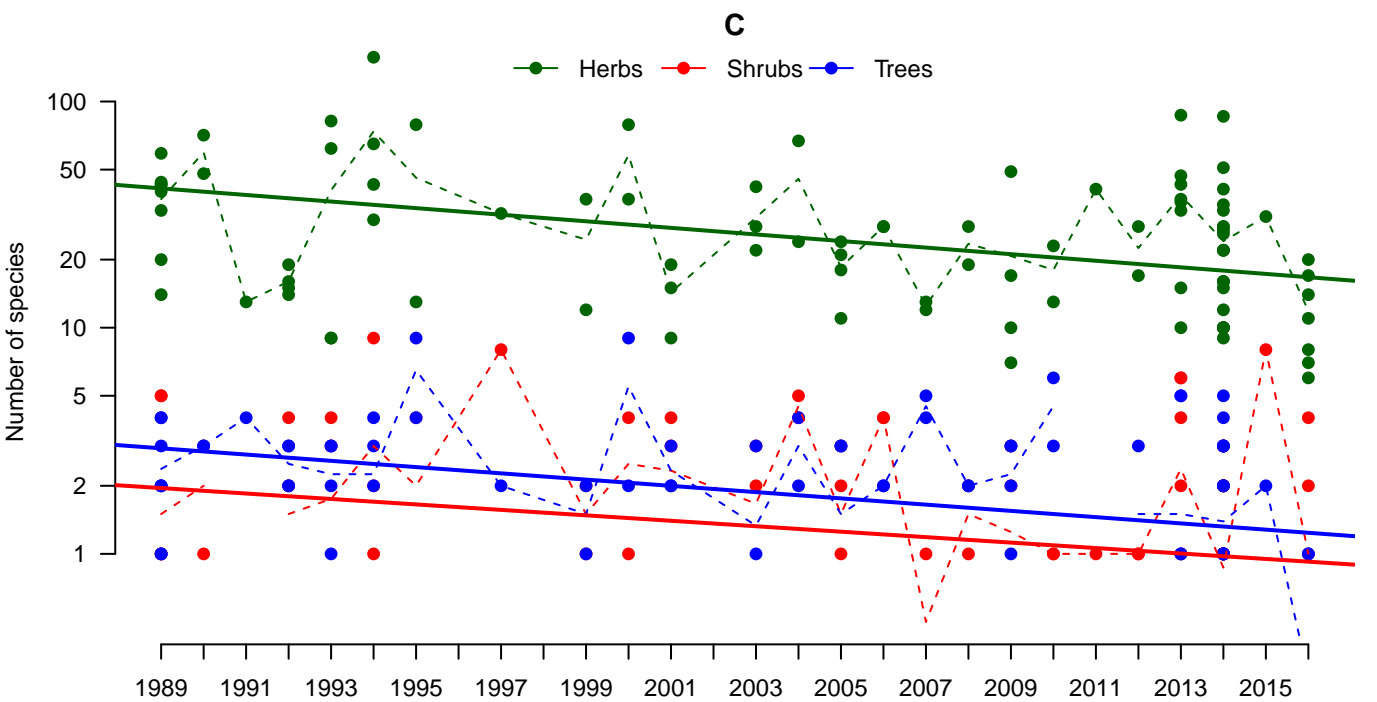

Supplement: S3 Fig — Mean land use in 1989–1994 (A) and 2012–2014 (B), based on aerial photograph analysis at 63 protected areas show a decrease of arable land and an increase in forested area over the past 25 years. (C) Changes in plants species richness for herbs (black) shrubs (red) and trees (blue). Annual means as well as mean trends are depicted in the corresponding colors. Linear trends are based on generalized linear mixed effects models with a Poisson error distribution and a random intercept effect for location. Note, zero values for tree and shrub species not depicted. (PDF) [file pone.0185809.s008.pdf]

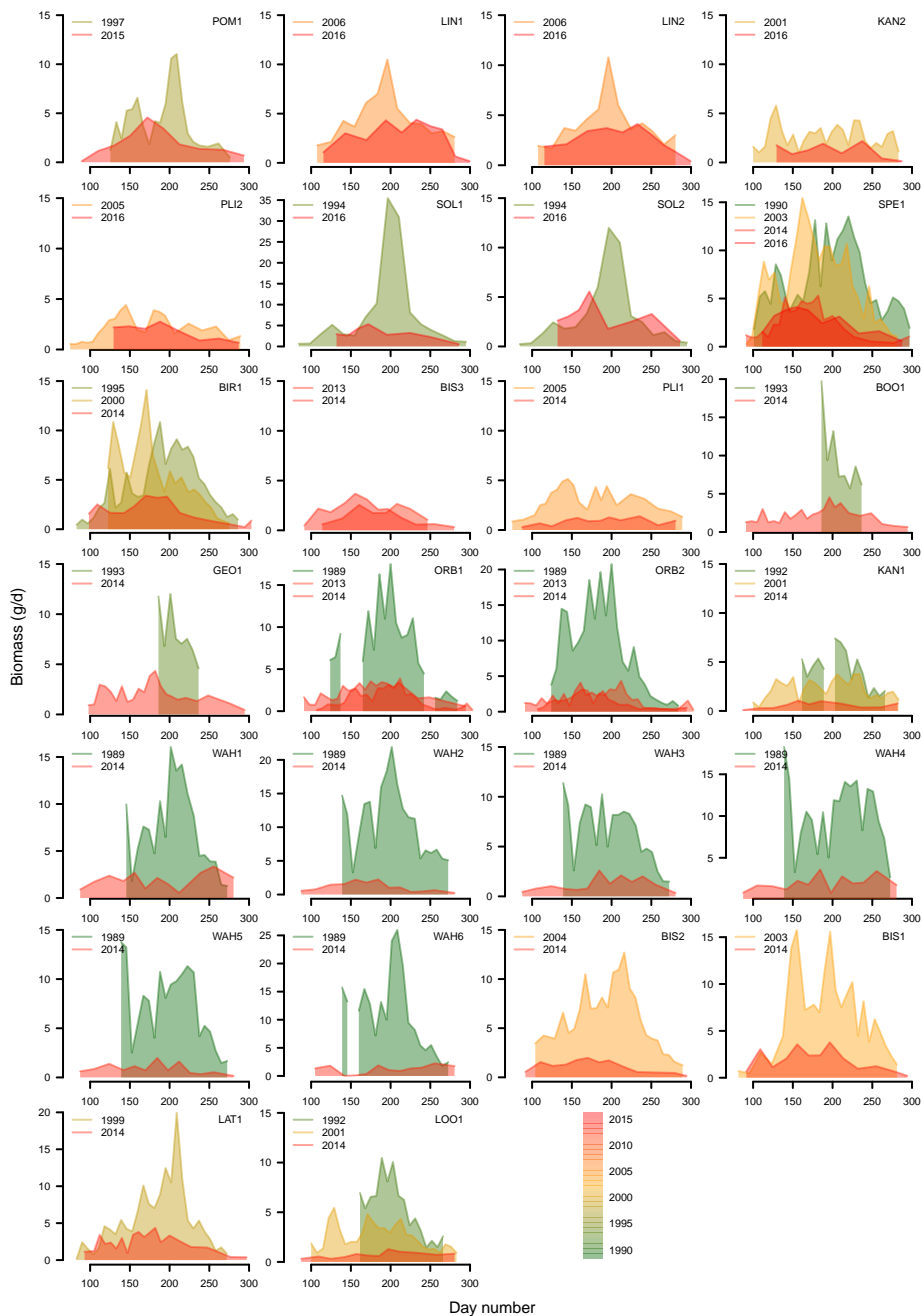

Supplement: S4 Fig — For each location, different colors represent different years, with time color-coded from green (1989) to red (2016). X-axis represents day number (January 1 = 0). (PDF) [file pone.0185809.s009.pdf]

**A**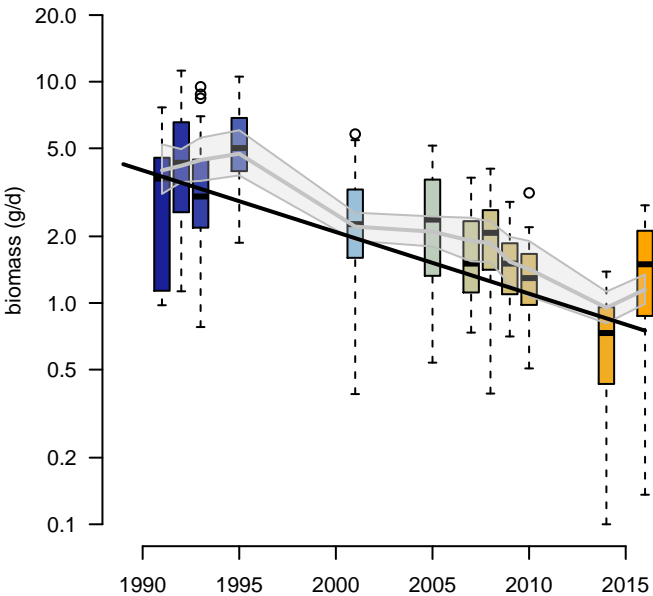**B**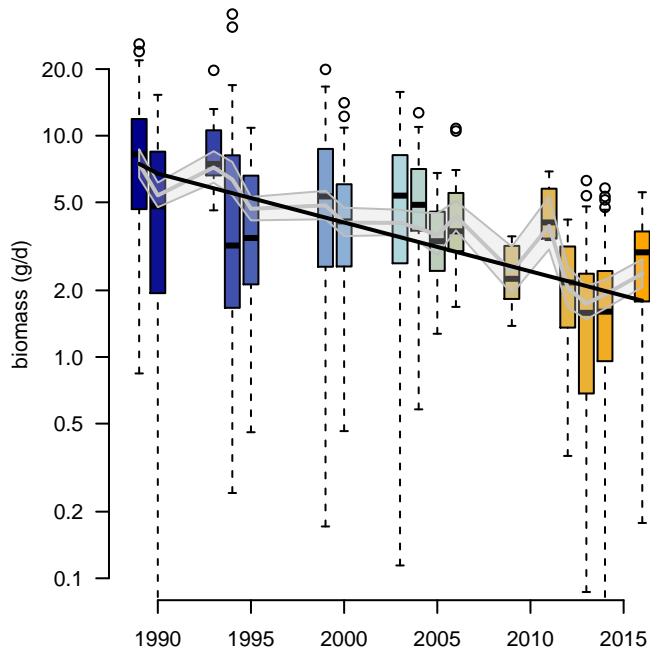

Supplement: S5 Fig — Boxplots depict the distribution of insect biomass pooled over all traps and catches in each year at trap locations in nutrient-poor heathland, sandy grassland, and dunes (A), and in nutrient-rich grasslands, margins and wasteland (B). Grey lines depict the fitted mean (+95% posterior credible intervals), while the black lines the mean estimated trend. Estimated annual decline amounts to 7.5%(6.6–8.4) for habitat cluster 1, as compared to 5.2% (4.8–5.5) habitat cluster 2. Models fitted independently for each habitat location. Color gradient in all panels range from 1989 (blue) to 2016 (orange). (PDF) [file pone.0185809.s010.pdf]
