# Supplementary material for: More than 75 percent decline over 27 years in total flying insect biomass in protected areas
Source: PLoS One. 2017 Oct 18;12(10):e0185809. doi: 10.1371/journal.pone.0185809 (PMC5646769; doi:10.1371/journal.pone.0185809)
Supplement: S1 Table — For each included variable, the corresponding coefficient posterior mean, standard deviation and 95% credible intervals are given. P-values are calculated empirically based on posterior distributions of coefficients. (PDF) [file pone.0185809.s011.pdf]

S1 Table. Posterior parameter estimates of the mixed effects model including weather variables. For each included variable, the corresponding coefficient posterior mean, standard deviation and 95% credible intervals are given. P-values are calculated empirically based on posterior distributions of coefficients.

**Variable**	**mean**	**sd**	**2.50%**	**97.50%**	**P**	
Intercept	1.947	0.086	1.772	2.112	<0.001	***
log(λ)	-0.058	0.002	-0.062	-0.054	<0.001	***
Day number	-0.127	0.029	-0.185	-0.069	<0.001	***
Day number^2	-0.437	0.030	-0.495	-0.378	<0.001	***
Temperature	0.298	0.023	0.251	0.343	<0.001	***
Precipitation	-0.062	0.034	-0.134	0.002	0.030	*
Wind speed	0.005	0.026	-0.046	0.057	0.418	
Frost days	-0.003	0.019	-0.041	0.034	0.430	
Winter Precipitation	0.025	0.019	-0.012	0.062	0.096	.
Habitat Cluster 2	0.298	0.090	0.117	0.479	0.001	***
Habitat Cluster 3	0.264	0.199	-0.121	0.649	0.091	.
Year × Day number	-0.001	0.001	-0.004	0.002	0.202	
Year × Day number^2	0.009	0.001	0.007	0.012	<0.001	***
σ_{site}	0.294	0.032	0.238	0.365		
v	0.884	0.009	0.866	0.902		
