# Supplementary material for: More than 75 percent decline over 27 years in total flying insect biomass in protected areas
Source: PLoS One. 2017 Oct 18;12(10):e0185809. doi: 10.1371/journal.pone.0185809 (PMC5646769; doi:10.1371/journal.pone.0185809)
Supplement: S2 Table — For each included variable, the corresponding coefficient posterior mean, standard deviation and 95% credible intervals are given. P-values are calculated empirically based on posterior distributions of coefficients. (PDF) [file pone.0185809.s012.pdf]

S2 Table. Posterior parameter estimates of the mixed effects model including land use variables and interactions. For each included variable, the corresponding coefficient posterior mean, standard deviation and 95% credible intervals are given. P-values are calculated empirically based on posterior distributions of coefficients.

**Variable**	**mean**	**sd**	**2.50%**	**97.50%**	**P**	
Intercept	2.551	0.146	2.248	2.832	<0.001	***
Year	-0.084	0.007	-0.098	-0.068	<0.001	***
Day number	-0.125	0.030	-0.183	-0.066	<0.001	***
Day number^2	-0.631	0.026	-0.681	-0.578	<0.001	***
Arable land	-0.848	0.191	-1.212	-0.467	<0.001	***
Forest	-0.529	0.204	-0.923	-0.125	0.006	**
Grassland	0.809	0.235	0.344	1.267	<0.001	***
Water	-0.475	0.212	-0.890	-0.067	0.011	*
Habitat Cluster 2	0.449	0.102	0.246	0.651	<0.001	***
Habitat Cluster 3	0.415	0.212	-0.005	0.838	0.026	*
Year × Day number	0.001	0.001	-0.002	0.004	0.215	
Year × Day number^2	0.011	0.001	0.009	0.014	<0.001	***
Year × Arable land	0.040	0.009	0.022	0.057	<0.001	***
Year × Forest	0.030	0.011	0.007	0.050	0.005	**
Year × Grassland	-0.062	0.014	-0.090	-0.033	<0.001	***
Year × Water	0.004	0.014	-0.024	0.032	0.399	
σ_{site}	0.306	0.035	0.245	0.380		
v	0.905	0.009	0.888	0.923		
