# Supplementary material for: More than 75 percent decline over 27 years in total flying insect biomass in protected areas
Source: PLoS One. 2017 Oct 18;12(10):e0185809. doi: 10.1371/journal.pone.0185809 (PMC5646769; doi:10.1371/journal.pone.0185809)
Supplement: S3 Table — For each included variable, the corresponding coefficient posterior mean, standard deviation and 95% credible intervals are given. P-values are calculated empirically based on posterior distributions of coefficients. (PDF) [file pone.0185809.s013.pdf]

S3 Table. Posterior parameter estimates of the mixed effects model including habitat variables. For each included variable, the corresponding coefficient posterior mean, standard deviation and 95% credible intervals are given. P-values are calculated empirically based on posterior distributions of coefficients.

**Variable**	**mean**	**sd**	**2.50%**	**97.50%**	**P**	
Intercept	2.385	0.188	1.999	2.768	<0.001	***
Year	-0.059	0.003	-0.065	-0.054	<0.001	***
Day number	-0.107	0.030	-0.167	-0.048	<0.001	***
Day number^2	-0.633	0.025	-0.681	-0.583	<0.001	***
Herb species	-0.087	0.049	-0.181	0.009	0.036	*
Tree species	0.105	0.033	0.043	0.170	0.001	***
Nitrogen	0.234	0.071	0.101	0.375	<0.001	***
pH	-0.051	0.061	-0.173	0.066	0.203	
Moisture	0.039	0.051	-0.061	0.139	0.220	
Light	0.185	0.041	0.106	0.267	<0.001	***
Ell. Temperature	-0.071	0.029	-0.128	-0.013	0.007	**
Habitat Cluster 2	0.350	0.159	0.036	0.654	0.014	*
Habitat Cluster 3	0.291	0.248	-0.203	0.781	0.120	
Year × Day number	0.001	0.002	-0.002	0.004	0.325	
Year × Day number^2	0.012	0.001	0.009	0.014	<0.001	***
σ_{site}	0.315	0.036	0.251	0.395		
v	0.909	0.009	0.891	0.927		
